# Increasing LFA-1 Expression Enhances Immune Synapse Architecture and T Cell Receptor Signaling in Jurkat E6.1 Cells

**DOI:** 10.3389/fcell.2021.673446

**Published:** 2021-07-23

**Authors:** Chiara Cassioli, Stefan Balint, Ewoud B. Compeer, James H. Felce, Alessandra Gamberucci, Chiara Della Bella, Suet Ling Felce, Jlenia Brunetti, Salvatore Valvo, Daniela Pende, Mario M. D’Elios, Lorenzo Moretta, Michael L. Dustin, Cosima T. Baldari

**Affiliations:** ^1^Department of Life Sciences, University of Siena, Siena, Italy; ^2^Kennedy Institute of Rheumatology, University of Oxford, Oxford, United Kingdom; ^3^Department of Molecular and Developmental Medicine, University of Siena, Siena, Italy; ^4^Department of Experimental and Clinical Medicine, University of Florence, Florence, Italy; ^5^Nuffield Department of Medicine, University of Oxford, Oxford, United Kingdom; ^6^Department of Medical Biotechnologies, University of Siena, Siena, Italy; ^7^IRCCS Ospedale Policlinico San Martino, Genova, Italy; ^8^Pediatric Hospital Bambino Gesù, Rome, Italy

**Keywords:** cSMAC, TCR signaling, supported lipid bilayer (SLB), Jurkat cell, LFA-1

## Abstract

The Jurkat E6.1 clone has been extensively used as a powerful tool for the genetic and biochemical dissection of the TCR signaling pathway. More recently, these cells have been exploited in imaging studies to identify key players in immunological synapse (IS) assembly in superantigen-specific conjugates and to track the dynamics of signaling molecules on glass surfaces coated with activating anti-CD3 antibodies. By comparison, Jurkat cells have been used only scantily for imaging on supported lipid bilayers (SLBs) incorporating laterally mobile TCR and integrin ligands, which allow to study synaptic rearrangements of surface molecules and the fine architecture of the mature IS, likely due to limitations in the assembly of immune synapses with well-defined architecture. Here we have explored whether upregulating the low levels of endogenous LFA-1 expression on Jurkat E6.1 cells through transduction with CD11a- and CD18-encoding lentiviruses can improve IS architecture. We show that, while forced LFA-1 expression did not affect TCR recruitment to the IS, E6.1 LFA-1^*high*^ cells assembled better structured synapses, with a tighter distribution of signaling-competent TCRs at the center of the IS. LFA-1 upregulation enhanced protein phosphotyrosine signaling on SLBs but not at the IS formed in conjugates with SEE-pulsed APCs, and led to the constitutive formation of an intracellular phosphotyrosine pool co-localizing with endosomal CD3ζ. This was paralleled by an increase in the levels of p-ZAP-70 and p-Erk both under basal conditions and following activation, and in enhanced Ca^2+^ mobilization from intracellular stores. The enhancement in early signaling E6.1 LFA-1^*high*^ cells did not affect expression of the early activation marker CD69 but led to an increase in IL-2 expression. Our results highlight a new role for LFA-1 in the core architecture of the IS that can be exploited to study the spatiotemporal redistribution of surface receptors on SLBs, thereby extending the potential of E6.1 cells and their derivatives for fine-scale imaging studies.

## Introduction

T cell immunological synapses (IS) are specialized cell-cell junctions between T cells and antigen presenting cells (APC) that are stable, demarcated by adhesion molecules and mediate vectoral cell–cell communication through a synaptic cleft (Dustin and Colman, 2002). Kupfer described the classical architecture of T-cell activation by APCs organized into supramolecular activation clusters (SMACs) with a ring of adhesive LFA-1-ICAM1 interactions forming the peripheral (p)SMAC and a central cluster of TCR in the central (c)SMAC (Monks et al., 1998), which together define the IS (Norcross et al., 1984; Dustin and Springer, 1998). The dynamic formation of ISs were first observed with supported lipid bilayers (SLB) presenting laterally mobile ICAM-1 and pMHC complexes, which enable formation of SMACs through a T cell autonomous process (Grakoui et al., 1999). Not only does LFA-1 mediate key interactions in the pSMAC, but the higher level of LFA-1 expression on memory versus naïve CD8 T cells is associated with more stable IS (Mayya et al., 2018), although this correlation has not been further tested for causality. In addition to lateral movement of LFA-1 and TCR in the plane of the plasma membrane, vesicular trafficking also plays a key role in IS formation (Onnis and Baldari, 2019; Mastrogiovanni et al., 2020). Early IS formation studies with cellular or SLB-based antigen presentation required use of primary T cells from transgenic mice. The use of readily available cell lines such as Jurkat would be advantageous for a number of reasons.

The acute T cell leukemia-derived Jurkat cell line has represented a robust tool to study T cell signaling, allowing for the biochemical and genetic identification of key players in the TCR signaling cascade (Abraham and Weiss, 2004). Jurkat cells activated by the Raji B lymphoblastoid cell line and staphylococcal superantigen E (SEE) have been useful, but do not provide fine resolution of IS structures (Blanchard et al., 2002). Jurkat cells have also been instrumental in reconstructing the dynamics of signaling molecules following TCR triggering when plated on glass surfaces coated with activating anti-CD3 antibodies through live imaging of transfected fluorescent reporters (Bunnell et al., 2001, 2002), which has been further refined with speckle microscopy (Kaizuka et al., 2007) and super-resolution methods (Fritzsche et al., 2017). This system led to the discovery that an F-actin ring is formed as T cells spread on surfaces with central clearance of F-actin (Bunnell et al., 2001). This central F-actin clearance with the formation of gaps in the mesh is a defining characteristic of the secretory domain that is critical for effector function (Ritter et al., 2015, 2017). A major intrinsic limitation of this approach is that, since the activating antibodies are immobile, the cells are unable to reorganize surface receptors and integrins to form the characteristic SMAC-based architecture of an IS and the method is only used to model early events in T-cell activation. The general strategy to efficiently incorporate laterally mobile antibodies into SLBs (Carrasco et al., 2004) made it possible to present laterally mobile anti-CD3 and ICAM1 to generate a Jurkat based IS model, however, in this setting TCR microclusters interspersed with LFA-1 clusters (Kaizuka et al., 2007, 2009; Murugesan et al., 2016; Chen et al., 2017), which are instead better separated with a more compact central TCR cluster in primary CD4^+^ T cells (Grakoui et al., 1999; Varma et al., 2006; Kumari et al., 2015).

Here, we have sought to improve IS architecture using an engineered Jurkat subline. Several clones have been derived from the original Jurkat line (Table 1). The most widely used to dissect TCR signaling is clone E6.1 (Weiss et al., 1984), from which subclones lacking individual TCR/CD3 components, integrin subunits or signaling molecules, such as Lck, ZAP-70, LAT, SLP-76, PLCγ, or CARMA-1, have been derived by mutagenesis (Abraham and Weiss, 2004). The basis for the less defined architecture of the IS formed by Jurkat E6.1 cells is not clear, but could include low levels of the integrin LFA-1 (CD11a/CD18) or associated co-factors such as talin and paxillin (Harburger and Calderwood, 2009). This limits the suitability of Jurkat E6.1 cells to track the dynamics of protein reorganization during IS assembly using SLB-based live imaging. Here we investigate whether increasing LFA-1 expression in Jurkat E6.1 cells can improve the architecture of ISs in the SLB setting. The results show that forcing increased LFA-1 surface expression by lentiviral transduction enhanced TCR segregation to the center of the cSMAC and local phosphotyrosine signaling. Forced LFA-1 expression did not enhance either the efficiency of TCR or tyrosine phosphoprotein accumulation at the IS formed by E6.1 cells in the setting of SEE-specific conjugates, but improved their ability to signal in response to TCR engagement, leading to increased IL-2 expression. Additionally, forced LFA-1 expression enhanced basal TCR signaling and accumulation of tyrosine phosphoproteins at CD3ζ^+^ endosomes. The results highlight a new role for LFA-1 in the core architecture of the IS formed by E6.1 cells that can be exploited to study the spatiotemporal redistribution of surface receptors on SLBs, thereby extending the potential of E6.1 cells and their derivatives for the study of T cell autonomous IS formation.

**TABLE 1:** Jurkat cell genealogy.

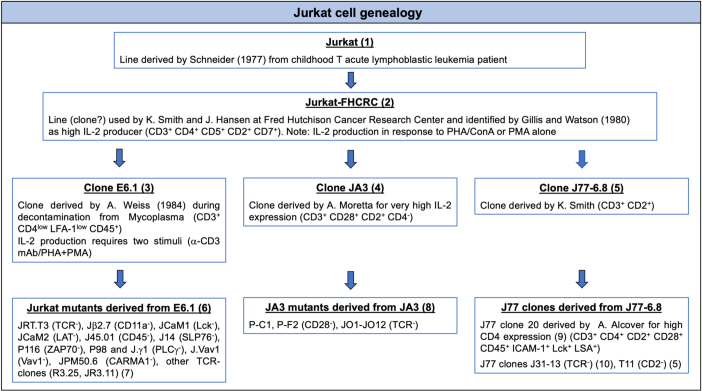

*(1) Schneider et al. (1977) Int J Cancer 19, 621.6*

*(2) Gillis and Watson (1980) J Exp Med 152, 1709-1719 (mentions gift Jurkat-FHCRC_w/o ref)*

*(3) Weiss et al. (1984) J Immunol 133, 123-128*

*(4) Moretta et al. (1985) J Exp Med 162, 823-838*

*(5) Moingeon et al. (1988) J Exp Med 168, 2077-2090 (mentions gift J77-6.8 w/o ref and generation of clones)*

*(6) Abraham and Weiss (2004) Nat Rev Immunol 4, 301-308*

*(7) Rubin et al. (2000) Scand J Immunol 52, 173-183*

*(8) Moretta et al. (1987) Proc Natl Acad Sci USA 84, 1654-1658*

*(9) Alcover et al. (1988) EMBO J 7, 1973-1977*

*(10) Danielian et al. (1992) Eur J Immunol 22, 2915-2921*

## Materials and Methods

### Cells and Lentiviral Transduction

Cells lines included Jurkat T cell clones E6.1 (Weiss et al., 1984) (obtained from ATCC) and JA3 (Moretta et al., 1985), and Raji B cells. A E6-1 line expressing high levels of LFA-1 was obtained by transduction with lentiviruses encoding LFA-1 subunits CD11a and CD18. Expression vectors for CD11a and CD18 were generated by insertion of their respective full-length genes into the lentiviral expression vector pHR. Genes were synthesized including terminal STOP codons and flanked by a 5′ *Mlu*I restriction exonuclease site and GCCACC Kozak sequence, and a 3′ *Not*I restriction site (GeneArt Gene Synthesis, ThermoFisher Scientific). Fragments were inserted into pHR vector via *Mlu*I + *Not*I restriction digestion and ligation by T4 DNA ligase. Lentiviruses were produced in HEK 293T cells at 3 × 10^5^/ml, 2 ml/well in 6-well plates in DMEM (#11960044) + 10% FBS (#15595309), 1% Glu (#25030-024), 1% Pen/Strep (#15140-122), all from Thermo Scientific. GeneJuice transfection was performed with vectors pHR-CD11a and pHR-CD18, pP8.91, and pMD.G. After 3 days of culture, supernatant was removed, spun at 500g for 5 min and passed through a 0.45 μm syringe filter. This virus solution was mixed with polybrene and spun down with Jurkat cells at 800 g for 90 min (RT, dec 6, acc 4). Cells were grown for >48 h at 37°C, 5% CO_2_ and passaged twice before stocks were frozen. Transduction efficiency was near 100% such that no selection was necessary. Experiments were performed with E6.1 LFA1^*high*^ cells from two transductions and the transduced cells were not used more than 10 passages. We used the validated ATCC E6.1 as a control for experiments in this paper. We note that E6.1 cells transduced with LifeAct-GFP (Fritzsche et al., 2017) and CXCR4-HaloTag (Felce et al., 2020) using the same lentiviral system showed no change in LFA-1 expression or IS organization compared to the untransduced ATCC E6.1 cells (unpublished observations).

Cells were cultured in RPMI 1640 medium (Life Technologies, #31870074) supplemented with 10% (vol/vol) FBS, 2 mM L-glutamine and 50 U/ml of Penicillin-Streptomycin at a max density of 1.5 × 10^6^/ml.

### RNA-seq Analysis

Raw counts for publicly deposited RNA-seq datasets (as used in Felce et al., 2021; Jurkat E6.1: GEO: GSE145453, Expression Atlas: E-MTAB-2706, GEO: GSE93435; Primary T cells: GEO: GSE122735, NCBI SRA: SRP026389, Expression Atlas: E-MTAB-3827) were normalized for gene length and *ACTB* mRNA counts. Fold difference was calculated as the mean normalized count in Jurkat samples relative to each primary T cell sample.

### Flow Cytometry

To assess surface expression of CD11a and CD3ε on Jurkat cells, 0.25 × 10^6^ cells/sample were washed and blocked with 2% FBS/PBS for 30 min at 4°C and then stained with anti-CD3ε-Alexa Fluor 488 (BioLegend, #317310) and anti-CD11a (ThermoFisher, #MA11A10) conjugated in house with Alexa Fluor 647 (ThermoFisher, #A20006), at 10 μg/mL for 30 min at 4°C. Finally, cells were washed three times in 2% FBS/PBS, fixed in 2% PFA/PBS, analyzed by LSRFortesa (BD Biosciences) with BD FACSDiva software and plotted using FlowJo version 10.

Flow cytometric analysis of surface CD69 was carried out using FITC anti-CD69, clone FN50 (BioLegend, #310904) at 1 μg/ml for 30 min at 4°C. Samples were analyzed with Guava Easy Cyte Cytometer (Millipore) and plotted using FlowJo version 6.1.1.

IL-2 intracellular staining flow cytometry was carried out using APC-labeled anti-human IL-2, clone MQ1-17H12 (BioLegend, #500310), at 0.125 μg/5 × 10^5^ cells. Samples were analyzed using a Becton Dickinson FACS CANTO II with BD FACSDiva 6.0 software.

For all experiments unstained cells and isotype controls were performed for background correction and gating.

### SLBs Preparation and Use

Planar Supported Lipid Bilayers (SLBs) were formed as previously described (Saliba et al., 2019). In brief, glass coverslips were cleaned with piranha solution (30% H_2_O_2_ and 70% H_2_SO_4_), rinsed extensively, dried, negatively charged through plasma cleaning, and assembled with a six-channel sticky-Slide VI 0.4 (Ibidi, #80608). SLBs were formed by filling each channel with a suspension of small unilamellar vesicles composed of 1,2-dioleoyl-*sn*-glycero-3-[*N*-(5-amino-1-carboxypentyp) succinyl] (12.5% mol) and 1,2-dioleoyl-*sn*-glycero-3-phosphoethanolamine-*N*-cap biotinyl (0.05% mol) in 1,2-dioleoyl-*sn*-glycero-3-phosphocholine at a total lipid concentration of 0.4 mM (Avanti Polar Lipids). SLBs were blocked with 5% casein (Sigma-Aldrich) in PBS containing 100 μM NiSO_4_. His-tagged proteins were incubated on bilayers for additional 20 min to obtain histidine-tagged–conjugated mouse ICAM1 at 200 molecules/μm^2^, histidine-tagged–conjugated UCHT1-Fab′ fragments at 30 molecules/μm^2^, and histidine-tagged–conjugated human CD80 at 100 molecules/μm^2^. Unbound proteins were flushed out with HEPES Buffered Salin Solution supplemented with 0.1% Human Serum Albumin (Calbiochem). Protein concentrations required to achieve desired densities on bilayers were calculated from calibration curves constructed from flow-cytometric measurements of bead-SLB, compared with reference beads containing known numbers of the appropriate fluorescent dyes (Bangs Laboratories). Bilayers were continuous liquid disordered phase as determined by fluorescence recovery after photobleaching with a 10 μm-bleach spot on an FV1200 confocal microscope (Olympus).

Jurkat cells were incubated at 37°C on SLB containing fluorescently labeled ICAM1-Alexa Fluor-405 and UCHT1-Fab’-CF568 and unlabeled CD80. After 15 min of incubation cells were fixed with 4% electron microscopy grade formaldehyde in PBS for 30 min at RT and washed three times with PBS. For intracellular staining, fixed cells were permeabilized and blocked with 0.01% Triton X-100 (Sigma-Aldrich, #X100-5ML) in blocking buffer (5% BSA/PBS) for 1 h at RT, washed three times with PBS, and stained with anti-CD3ζ Alexa Fluor-647 (SantaCruz, #SC-1239 AF647), anti-pTyr Alexa Fluor-488 (Biolegend, #309306) at 10 μg/mL in 5% BSA/PBS for 1 h at RT followed by three times washing with PBS. Cells were also stained with Alexa Fluor-405 Phalloidin (Thermo Fisher Scientific, # A30104) to highlight actin cytoskeleton.

### Conjugate Formation

Conjugates between Jurkat cells and SEE-pulsed Raji B cells were carried out as previously described (Finetti et al., 2009). Raji cells were pulsed for 2 h with 10 μg/ml SEE (Toxin Technology, Sarasota, FL, United States) and labeled with 10 μM Cell Tracker Blue (Molecular Probes) for the last 20 min. Conjugates between T cells and unpulsed B cells were used as negative controls. SEE-pulsed or unpulsed Raji B cells were mixed with Jurkat T cells (1:1) and conjugates analyzed 15 min after their formation. Samples were allowed to adhere for 15 min on poly-L-lysine (Sigma-Aldrich)-coated wells of diagnostic microscope slides (ThermoFisher Scientific), then fixed by immersion in methanol for 10 min at -20°C. Following fixation, samples were washed in PBS and incubated with anti-pTyr (Cell Signaling, #8954) at 10 μg/mL and anti-CD3ζ at 15 μg/mL (SantaCruz, #sc-1239) in PBS 1X overnight at 4°C. After washing in PBS, samples were incubated for 45 min at room temperature with anti-rabbit Alexa-Fluor-488- and anti-mouse Alexa-Fluor-555-labeled secondary antibodies (ThermoFisher Scientific, #A11008 and #A211422, respectively).

### Fluorescence Microscopy

TIRFM was performed on an Olympus IX83 inverted microscope equipped with a 4-line (405, 488, 561, and 640 nm laser) illumination system. The system was fitted with an Olympus UApON 150 × 1.45 NA objective, and a Photomertrics Evolve delta EMCCD camera to provide Nyquist sampling. Analysis of TIRFM images was performed with ImageJ (National Institute of Health). Mean fluorescence intensities of ICAM-1, CD3ε, CD3ζ, pTyr and phalloidin were calculated as the sum of intensities in each pixel in the cell spreading area divided by the spreading area of the corresponding cell. The spreading area was determined by thresholding the IRM (Interference Reflection Microscopy) images of each cell.

Intensity compactness of CD3ε clusters represents how compact the intensity signal from each CD3ε cluster is within the defined area. If the CD3ε clusters are concentrated around the center of the defined area, the intensity compactness is closer to 1. If the CD3ε clusters are more sparsely and heterogeneously distributed within the defined area, the value goes toward 0. The area to calculate the intensity of compactness was defined by the cSMAC of Jurkat cells. The ImageJ plugin to calculate the intensity compactness was kindly provided by Prof. Jérémie Rossy (Biotechnology Institute Thurgau, University of Konstanz, Switzerland).

Confocal microscopy imaging of Jurkat cells on SLB was carried out on a Zeiss LSM980 (Zeiss, Germany) using a 63 × 1.40 NA oil immersion objective. Cells were stained as for TIRF microscopy and 3D confocal imaging was carried out at 200 nm z-steps. The orthogonal views and 3D reconstruction of images was performed using ImageJ (National Institute of Health).

Confocal microscopy of cell–cell conjugates was carried out on a Zeiss LSM700 (Zeiss, Germany) using a 63x/1.40 oil immersion objective. Images were acquired with pinholes opened to obtain 0.8 μm-thick sections. Detectors were set to detect an optimal signal below the saturation limits. Images were processed with Zen 2009 image software (Carl Zeiss, Jena, Germany). The quantitative co-localization analysis of pTyr with CD3ζ in E6.1 LFA-1^*high*^ cells was performed on median optical sections using ImageJ and JACoP plug-in to determine Manders’ coefficient M1 (Manders et al., 1992). Scoring of conjugates for pTyr clustering at the IS was based on the concentration of the respective staining solely at the T-cell:APC contact. The recruitment index of pTyr was calculated as either the relative fluorescence at the T-cell:APC contact site compared to the mean fluorescence of three membrane regions with the same area outside of the contact (Figure 3B) or the relative fluorescence at the T-cell:APC contact site compared to the entire cell, either excluding (Figure 3D) or including (Figure 3C) the fluorescence of the constitutive intracellular pTyr pool. 3D confocal imaging of conjugates was carried out on a Leica TCS SP8 (Leica, Germany) at 220 nm z-steps. The orthogonal views and 3D reconstruction of images were performed using ImageJ (NIH).

**FIGURE 1:**
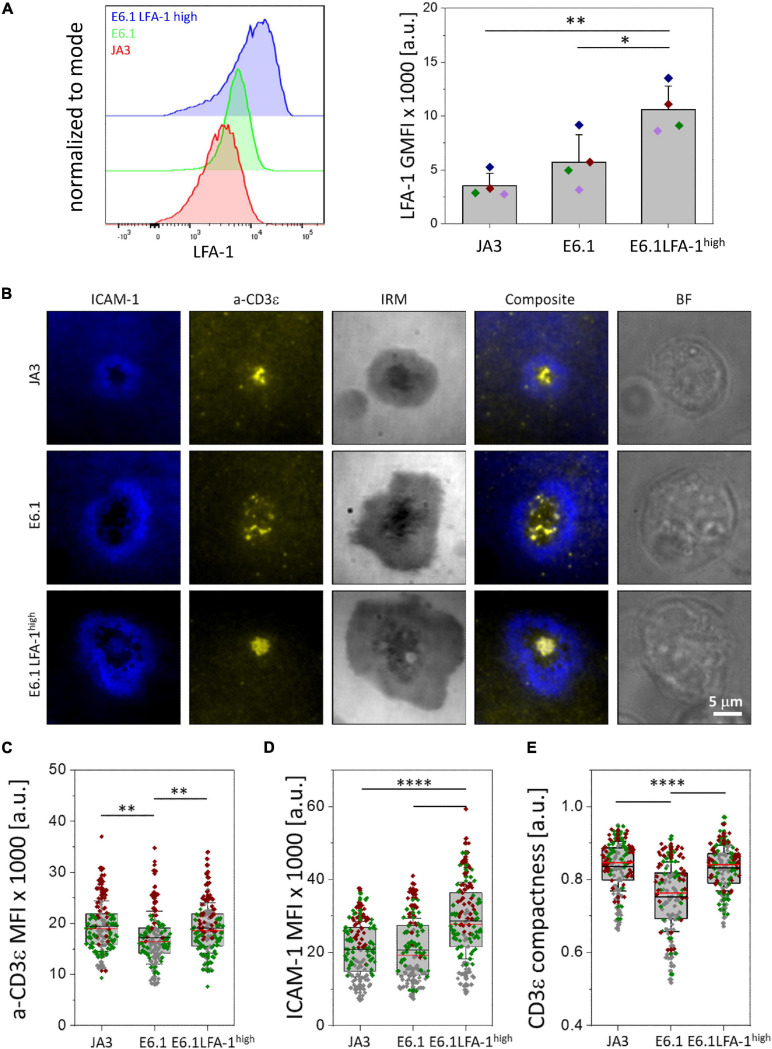
Increasing LFA-1 expression in E6.1 cells improves TCR segregation to the cSMAC center. **(A)** Flow cytometric analysis of surface expression of LFA-1 (CD11a) on JA3, E6.1 and E6.1 LFA-1^*high*^ Jurkat cells. Representative FACS plots of LFA-1 are shown. The histogram shows GMFI of LFA-1 (mean ± SD). **(B)** Representative TIRFM images of E6.1, E6.1 LFA-1^*high*^ or JA3 Jurkat cells interacting with activating [ICAM1-AF405 (blue) + anti-CD3ε UCHT1-CF568 Fab’ (yellow)] SLBs for 15 min. IRM, interference reflection microscopy; BF, bright field. Scale bar, 5 μm. **(C,D)** Quantification of the corresponding mean fluorescent intensity (MFI) of anti-CD3ε **(C)** and ICAM-1 **(D)** from the different Jurkat lines as in **(B)**. **(E)** Quantification of the CD3ε compactness at the cSMAC from the experiment in **(B)**. Horizontal lines and error bars represent mean ± SD (black line) and median (red line). Gray boxes represent 25-75 percentile. Data are from minimum of 120 cells from three independent experiments; each dot represents a cell; each color represents an independent experiment. One-way analysis of variance (ANOVA) with Tukey’s *post hoc* test. Only significant differences are shown. ^∗^*p* < 0.05; ^∗∗^*p* < 0.01; ^****^p < 0.0001.

**FIGURE 2:**
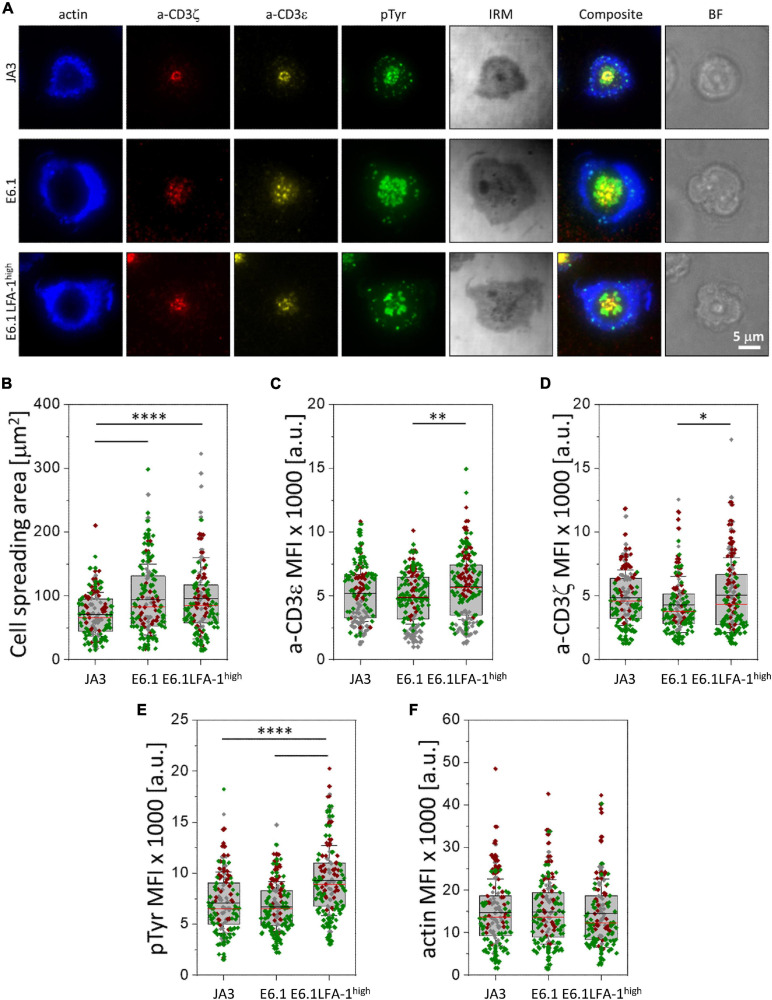
Increasing LFA-1 expression in E6.1 cells improves phosphotyrosine signaling at the cSMAC. **(A)** Representative TIRFM images of E6.1, E6.1 LFA-1^*high*^ or JA3 Jurkat cells interacting with activating [ICAM1 + anti-CD3ε UCHT1-CF568 Fab’ (yellow)] SLB for 15 min. Cells were permeabilized and stained with directly conjugated primary antibodies against anti-CD3ζ-AF647 (red), anti-phosphotyrosine [pTyr-AF488 (green)]; the cell actin cytoskeleton was labeled with phalloidin-AF405 (blue). IRM, interference reflection microscopy; BF, bright field. Scale bar, 5 μm. **(B)** Quantification of cell spreading area of different Jurkat cell lines on activating SLB as in **(A)**. Corresponding mean fluorescent intensity (MFI) of anti-CD3ε **(C)**, anti-CD3ζ **(D)**, anti-pTyr **(E)** and actin **(F)** from different Jurkat cell lines as in **(A)**. Horizontal lines and error bars represent mean ± SD (black line) and median (red line). Gray box represents 25–75 percentile. Data are from minimum of 130 cells from three independent experiments; each dot represents a cell; each color represents an independent experiment. One-way analysis of variance (ANOVA) with Tukey’s *post hoc* test. Only significant differences are shown. ^∗^*p* < 0.05; ^∗∗^*p* < 0.01; ^****^p < 0.0001.

**FIGURE 3:**
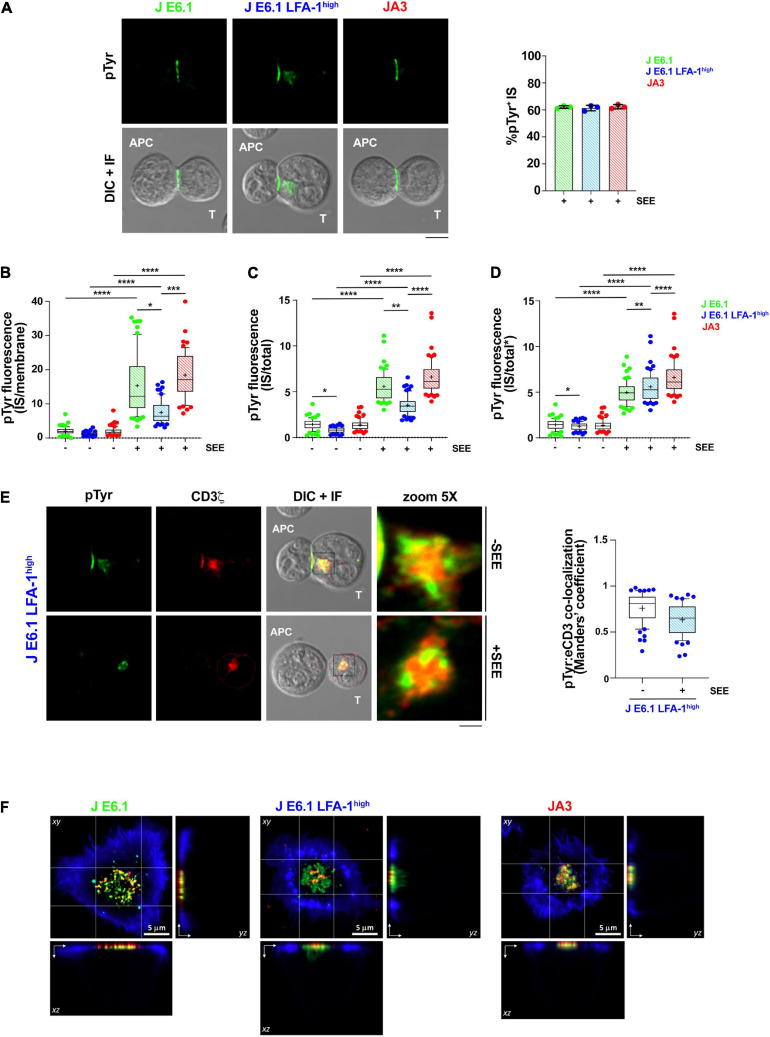
Increasing LFA-1 expression in E6.1 cells does not improve phosphotyrosine signaling at the IS in conjugates with SEE-loaded APCs. **(A)** Immunofluorescence analysis of tyrosine phosphoproteins (pTyr) in 15-min conjugates of E6.1, E6.1 LFA-1^*high*^ or JA3 Jurkat cells and SEE-pulsed Raji cells (APC). Representative images are shown. The histogram shows the percentage (mean ± SD) of conjugates with pTyr staining at the T-cell:APC contact (right; unpaired Student’s *t*-test; *n* > 40 conjugates/sample from three independent experiments). **(B)** Quantification of the relative mean fluorescence intensity (MFI) of anti-pTyr at the IS membrane versus the mean of anti-pTyr MFIs measured at three different membrane regions of the same size outside of the T-cell:APC contact (mean ± SD; Kruskal–Wallis test; *n* ≥ 20 conjugates/sample from three independent experiments). Graph boxes represent 10–90 percentile and the mean is shown as “+.” **(C)** Quantification of the relative mean fluorescence intensity (MFI) of anti-pTyr at the IS membrane versus the MFI of the whole T cell (total) (mean ± SD; Kruskal–Wallis test; *n* ≥ 20 conjugates/sample from three independent experiments). Graph boxes represent 10–90 percentile and the mean is shown as “+.” **(D)** Quantification of the relative mean fluorescence intensity (MFI) of anti-pTyr at the IS membrane versus the MFI of the whole T cell excluding the intracellular pTyr pool in E6.1 LFA-1^*high*^ cells (total*) (mean ± SD; Kruskal–Wallis test; *n* ≥ 20 conjugates/sample from three independent experiments). Graph boxes represent 10–90 percentile and the mean is shown as “+.” **(E)** Immunofluorescence analysis of 15-min conjugates of E6.1, E6.1 LFA-1^*high*^ or JA3 cells and Raji cells (APC) in the presence or absence of SEE. Cells were co-stained with anti-pTyr and anti-CD3ζ antibodies. Representative images of E6.1 LFA-1^*high*^ cells are shown (representative xy images of E6.1 and JA3 cells are shown in Supplementary Figure 5A, orthogonal views from 3D confocal images are shown in Supplementary Figure 5B and 3D reconstructions of representative z-stacks in videos 1–3). The histogram shows the quantification (mean ± SD) of the co-localization of intracellular pTyr^+^ pool and endosomal CD3ζ (eCD3) in E6.1 LFA-1^*high*^ cells (Manders’ coefficient) (mean ± SD; Kruskal–Wallis test; *n* ≥ 20 conjugates/sample from three independent experiments). Graph boxes represent 10–90 percentile and the mean is shown as “+.” Scale bar, 5 μm. Only significant differences are shown. *p < 0.05; **p < 0.01; ***p < 0.001; ****p < 0.0001. **(F)** Representative xy and orthogonal views from 3D confocal images of E6.1, E6.1 LFA-1^ high^ or JA3 Jurkat cells interacting with activating [ICAM1 + anti-CD3ε UCHT1-CF568 Fab’ (red)] SLB for 15 min (3D reconstructions of representative z-stacks are shown in Supplementary Videos 4–6). Cells were permeabilized and stained with directly conjugated primary antibodies against anti-phosphotyrosine [pTyr-AF488 (green)] and the cell actin cytoskeleton was labeled with phalloidin-AF405 (blue). Scale bar, 5 μm.

### Cell Activation for Immunoblot and Flow Cytometric Analysis of Activation Markers

For immunoblot experiments 12-well plates were coated with 5 μg/ml of anti-CD3ε mAb, clone OKT3 (BioLegend, #317302) and 1 μg/ml of ICAM1 Fc (R&D System, #720-IC) in PBS 1X overnight at 4°C or for 2 h at 37°C, followed by extensive rinse with PBS 1X. Cells (2.5 × 10^6^ cells/sample) were pelleted, resuspended in serum-free RPMI-1640 at the density of 1 × 10^6^ cells/ml, then seeded on non-activating (ICAM-1) or activating (ICAM-1 + anti-CD3ε) plates and incubated for 5 or 20 min at 37°C, 5% CO_2_. Unstimulated samples were included as negative controls.

For the quantification of T-cell activation Jurkat cells (0.15 × 10^6^ cells/sample) seeded on either plate-bound anti-CD3ε or anti-CD3ε + ICAM-1 and incubated for 16 h at 37°C, 5% CO_2_. Surface CD69 was quantified by flow cytometry. T cells activated with 100 ng/ml PMA and 500 ng/ml A23187 were used as positive control. For IL-2 measurements, 10^6^ cells were activated on plate-bound anti-CD3ε mAb, clone OKT3 (BioLegend, #317302) and anti-CD28 mAb, clone CD28.2, all at 5 μg/ml in PBS 1X (BioLegend, #302902) for 6 h. After 1 h incubation, the protein transport inhibitor cocktail containing brefeldin A and monensin (eBioscience, #00-4980-93) was added (2 μl/ml). Membrane permeabilization was done by BD cytofix/cytoperm fixation/permeabilization kit (BD Bioscience, #554714) according to the manufacturer’s protocol prior to processing samples for flow cytometry.

### Immunoblots

Cells (2.5 × 10^6^ cells/sample) were pelleted, washed twice in ice-cold PBS and lysed in 25 μl lysis buffer (1% Triton X-100 in 20 mM Tris-HCl, pH 8.0, 150 mM NaCl) in the presence of Protease Inhibitor Cocktail Set III (Calbiochem) and 0.2 mg of the phosphatase inhibitor sodium orthovanadate (Sigma-Aldrich). Quantification of total protein content was carried out using the Quantum Protein Assay Kit according to manufacturer’s protocol (EuroClone). Post-nuclear supernatants were denatured in 4X Bolt^TM^ LDS Sample Buffer (Invitrogen) supplemented with 10X Bolt^TM^ LDS Sample Buffer (Invitrogen) for 5 min at 100°C. Proteins were subjected to SDS-PAGE on Bolt^TM^ Bis-Tris Mini Protein Gels (Invitrogen) and transferred to nitrocellulose (GE HealthCare) under wet conditions. Blocking was performed in 5% non-fat dry milk in PBS 0.02% Tween-20. Membranes were incubated with anti-p-ZAP70 Y319 (Cell Signaling, #2701) and anti-p-Erk1/2 T202/Y204 (Cell Signaling, #9101) primary antibodies at RT followed by secondary horseradish-peroxidase (HRP)-conjugated antibodies for 45 min at RT. Secondary antibodies were detected by using SuperSignal^TM^ West Pico Plus Chemiluminescent Substrates (ThermoFisher Scientific). Membranes were stripped with ReBlot Plus Mild Antibody Stripping Solution 10X (Chemicon) and reprobed with anti-ZAP70 (Merck Millipore, #05-253) and anti-Erk2 (SantaCruz, #sc-1647) control antibodies. Blots were scanned using a laser densitometer (ET-M2170; EPSON, Suwa, Yapan) and quantified using ImageJ (NIH).

### [Ca^2+^] Measurements

Jurkat cells were loaded with the fluorescent calcium indicator Fura- 2 acetoxymetyl ester (Fura-2/AM) to evaluate changes of cytosolic free calcium concentration as described (Gamberucci et al., 1994). Briefly, after Fura-2-AM (3 μM) loading for 30 min at RT, the cells were maintained in medium (140 mM NaCl, 5.4 mM KCl, 1 mM MgCl2, 1 mM CaCl_2_, 10 mM glucose, 15 mM Hepes buffer, pH 7.4). Aliquots were rapidly centrifuged and resuspended in fresh nominally Ca^2+^ free medium at the density of 2 × 10^6^ cells/1 ml: cytosolic Ca^2+^ basal levels were obtained by adding Na-ethylene glycol bis(B-amino ethyl ether)-N,N,N0,N0 tetraacetic acid (EGTA) to nominally Ca^2+^ free medium to eliminate possible calcium traces; reticular Ca^2+^ was evaluated after depletion of the stores with anti-CD3ε mAb, clone OKT3 at 1.3 μg/ml (BioLegend, #317302) followed by cross-linking with anti-mouse IgG at 10 μg/ml. The subsequent Ca^2+^ influx through the plasma membrane channels was evaluated by re-adding 1.7 mM CaCl_2_.

Fura-2 fluorescence was measured using a Varian Cary Eclipse fluorescence spectrophotometer (Palo Alto, CA, United States) (excitation wavelengths, 340 and 380 nm; emission, 510 nm) equipped with magnetic stirring and temperature control set at 35°C. At the end of each experiment, digitonin (20 mg/ml) and EGTA (10 mM) (Sigma-Aldrich) were added in order to measure maximal (Rmax) and minimal (Rmin) ratio (340/380) fluorescence values, respectively. The equation [Ca^2+^]_i_ = (R-R_*min*_)/(R_*max*_-R)Sf^∗^Kd was used to convert the Fura-2 ratio values to intracellular calcium concentrations.

Total [Ca^2+^]_i_ release over time was quantified by measuring the Area Under the Curve (AUC) that represents the area enclosed under the curve of the [Ca^2+^]_i_ versus the time for 4 min after OKT3 addition; AUC was calculated using GraphPad Prism version 7.00 (La Jolla, CA, United States).

### Statistics

The number of repeats and the number of cells analyzed is specified in each figure legend. Statistical analyses were performed using GraphPad Software (La Jolla, CA, United States). Pairwise or multiple comparisons among values with normal distribution were carried out by using Student’s *t*-test (paired or unpaired) and one-way ANOVA with Tukey’s *post hoc* test, whereas values without Gaussian distribution were analyzed with Mann–Whitney test or Kruskal–Wallis test. Statistical significance was defined as: ns *p* > 0.05, ^∗^*p* ≤ 0.05, ^∗∗^*p* ≤ 0.01, ^∗∗∗^*p* ≤ 0.001, ^****^p ≤ 0.0001.

## Results

### Forced LFA-1 Expression in E6.1 Cells Improves TCR Segregation to the cSMAC Center

Transcriptomic comparison of E6.1 cells against primary CD4^+^ T cells using RNA-seq (public datasets as used in Felce et al., 2021) revealed that primary T cells have 8.7 ± 5.5 times more *ITGAL* mRNA (encoding CD11a) and 7.1 ± 2.6 times more *ITG2B* mRNA (encoding CD18) than E6.1 cells. To investigate whether the amount of surface LFA-1 influences the core architecture of the IS formed by two most commonly used Jurkat cell lines, E6.1 (Weiss et al., 1984) and JA3 (Moretta et al., 1985), we first measured the surface expression levels of CD11a and CD3ε by flow cytometry. Surface expression levels of CD11a on both cell lines were comparable (Figure 1A and Supplementary Figure 1A). Both lines displayed comparable levels of surface CD3ε and showed the presence of a CD3ε^*low*^ population that was more abundant among JA3 cells (Supplementary Figure 1B).

To assess the bull’s eye pattern of the mature IS architecture, cells were plated on SLB containing an agonistic anti-CD3ε (UCHT1) Fab′ fragment labeled with CF568 and the LFA-1 ligand ICAM-1 directly conjugated with Alexa Fluor-405, and analyzed by total internal reflection fluorescence microscopy (TIRFM) (Figure 1B and Supplementary Figures 2A,B). Cells were plated for 15 min, a time point at which, in primary T cells, the TCR and LFA-1 have segregated into the cSMAC and pSMAC, respectively. Slightly higher levels of CD3ε were observed at the interface of JA3 cells compared to E6.1 cells with SLB, as measured by mean fluorescence intensity (MFI) of the UCHT1 signal at the SLB (Figure 1C). This small increase was due to the smaller cell spreading area of JA3 cells (Supplementary Figure 3). Of note, similar to E6.1 cells, JA3 cells formed an ICAM-1 ring, which can only be elicited in the presence of a strong TCR signal, indicating that the presence of a more abundant population of CD3ε^*low*^ among JA3 cells does not compromise their ability to assemble immune synapses. The levels of LFA-1 measured by mean fluorescence intensity of ICAM-1 on SLBs were not significantly different between E6.1 and JA3 (Figure 1D), supporting the results obtained by flow cytometry (Figure 1A and Supplementary Figure 1A). Hence both Jurkat cell lines have similar capability of CD3 transport that underlies TCR spatial organization and signaling at the IS (Onnis and Baldari, 2019). However, by assessing the images in Figure 1B and Supplementary Figures 2A,B, JA3 cells formed more compact and tighter CD3-enriched cSMACs within the ICAM-1-enriched ring compared to E6.1 cells. This was confirmed by measuring intensity compactness to quantify the distribution of CD3ε clusters within the cSMAC of E6.1 and JA3 cells (see section “Materials and Methods” for description). We observed significant differences between the two cell lines, with CD3ε clusters showing a lower compactness in E6.1 cells compared to JA3 cells (Figure 1E).

As mentioned above, primary T cells have approximately 9 times more LFA-1 than E6.1 Jurkat cells and are known to form compact CD3ε-enriched cSMAC (Demetriou et al., 2020). To directly address whether increasing LFA-1 levels could improve cSMAC formation in E6.1 cells, we transduced these cells with lentiviruses encoding human CD11a and CD18, generating the E6.1 LFA-1^*high*^ line. E6.1 LFA-1^*high*^ cells expressed higher levels of surface LFA-1 compared to both parental E6.1 cells (∼2-fold) and JA3 cells (∼3-fold), as assessed by flow cytometry (Figure 1A and Supplementary Figure 1A). CD3ε surface levels were comparable across Jurkat cell lines (Supplementary Figure 1B).

To analyze TCR compartmentalization at the cSMAC, E6.1 LFA-1^*high*^ cells were plated on SLBs as for E6.1 and JA3, and analyzed by TIRFM (Figure 1B and Supplementary Figure 2C). As expected, forced LFA-1 expression in E6.1 cells increased the amount of ICAM-1-engaged LFA-1 on SLBs (Figure 1D). E6.1 LFA-1^*high*^ cells formed a tight and compact CD3ε-enriched cSMAC within the ICAM-1-enriched ring (Figures 1B,E and Supplementary Figure 2C), with a similar cell spreading area (Supplementary Figure 3) but an increase in the levels of CD3ε at the interface with the SLB compared to E6.1 cells (Figure 1C). Together, these data demonstrate that LFA-1 enhances the efficiency of TCR recruitment to the cSMAC and facilitates the formation of the core IS architecture.

### Increasing LFA-1 Expression in E6.1 Cells Improves Phosphotyrosine Signaling at the IS Formed on Activating SLBs but Not in SEE-Specific Cell Conjugates

To understand whether the increased levels of LFA-1 can enhance signaling, we measured the accumulation of tyrosine phosphoproteins at the IS formed in the SLB setting. E6.1, E6.1 LFA-1^*high*^ and JA3 cells were plated on SLBs containing an agonistic anti-CD3ε (UCHT1) Fab’ fragment labeled with CF568 and the LFA-1 ligand ICAM-1 directly conjugated with Alexa Fluor-405 for 15 min and analyzed by TIRFM (Figure 2A and Supplementary Figure 4). After fixation and permeabilization, cells were stained with directly conjugated primary antibodies against anti-CD3ζ Alexa Fluor-647, anti-phosphotyrosine (pTyr) Alexa-Fluor-488, and phalloidin Alexa Fluor-405 to visualize the underlying actin cytoskeleton (Figure 2A and Supplementary Figure 4). Again, JA3 cells had smaller spreading areas (Figure 2B) with levels of CD3ε and CD3ζ accumulation comparable to E6.1 and E6.1 LFA-1^*high*^ cells, while accumulation was higher in E6.1 LFA-1^*high*^ cells compared to E6.1 cells (Figures 2C,D). Quantification of the TIRFM images revealed higher pTyr levels in E6.1 LFA-1^*high*^ cells compared to parental E6.1 (Figure 2E), likely due to their more compact cSMAC. PTyr signaling in E6.1 LFA-1^*high*^ cells was also more efficient compared to JA3 cells (Figure 2E). In all Jurkat lines the underlying actin cytoskeleton formed an actin ring, which is a marker of cell activation, and LFA-1 overexpression did not alter the amount of actin at the SLB contact (Figure 2F).

To further understand whether the ability of LFA-1 to influence the architecture of the IS in the SLB setting translates into enhanced signaling in cell-cell conjugates, we compared E6.1, E6.1 LFA-1^*high*^ and JA3, cells in the classical context of SEE-specific conjugates. Jurkat cells were mixed with SEE-pulsed Raji cells (used as APC) for 15 min and the accumulation of tyrosine phosphoproteins at the T-cell:APC interface was measured by confocal microscopy. All Jurkat lines showed a comparable proportion of pTyr-positive conjugates (Figure 3A and Supplementary Figure 5). Unexpectedly, as opposed to the SLB setting, the accumulation of tyrosine phosphoproteins at the synaptic membrane upon activation was lower in E6.1 LFA-1^*high*^ cells compared to E6.1 cells, both when calculating the ratio of the membrane pTyr signal versus the average of three similarly sized regions of the non-synaptic membrane, and when calculating the ratio of the membrane pTyr signal versus the total cellular pTyr signal (Figures 3B,C). This suggests that in the more complex setting of cell-cell conjugates other integrins or costimulatory receptors contribute to the accumulation of tyrosine phosphoproteins at the IS. Remarkably, as opposed to parental E6.1 cells, E6.1 LFA-1^*high*^ cells displayed a vesicular pTyr pool that localized just beneath the synaptic membrane in the majority of conjugates and was present also in the absence of SEE (Figure 3A and Supplementary Figure 5). The pTyr^+^ pool was not observed in JA3 cells, which displayed pTyr accumulation at the synaptic membrane comparable to E6.1 cells (Figure 3A and Supplementary Figures 5,6A). Of note, when the signal of the constitutive intracellular pTyr pool was subtracted from the total cellular pTyr signal, the accumulation of tyrosine phosphoproteins at the synaptic membrane of E6.1 LFA-1^*high*^ cells was not significantly different from E6.1 of JA3 cells (Figure 3D and Supplementary Videos 1–3). Interestingly, the vesicular pTyr^+^ pool unique to E6.1 LFA-1^*high*^ cells co-localized with the intracellular pool of CD3ζ (Figure 3E, Supplementary Figures 6A–C, and Supplementary Videos 1–3).

To better visualize the accumulation of tyrosine phosphoproteins at the IS of E6.1, E6.1 LFA-1^*high*^ and JA3 cells, we performed 3D confocal imaging on SLBs. Visualization of all three cell lines by their corresponding orthogonal views revealed the presence of the internal pTyr pool only in E6.1 LFA-1^*high*^ cells (Figure 3F and Supplementary Videos 4–6). Together, these results suggest that the redistribution of tyrosine phosphoproteins into a synaptic and subsynaptic pool in E6.1 LFA-1^*high*^ cells is a consequence of forced LFA-1 expression.

### Increasing LFA-1 Expression in E6.1 Cells Enhances TCR Signaling and IL-2 Expression

**FIGURE 4:**
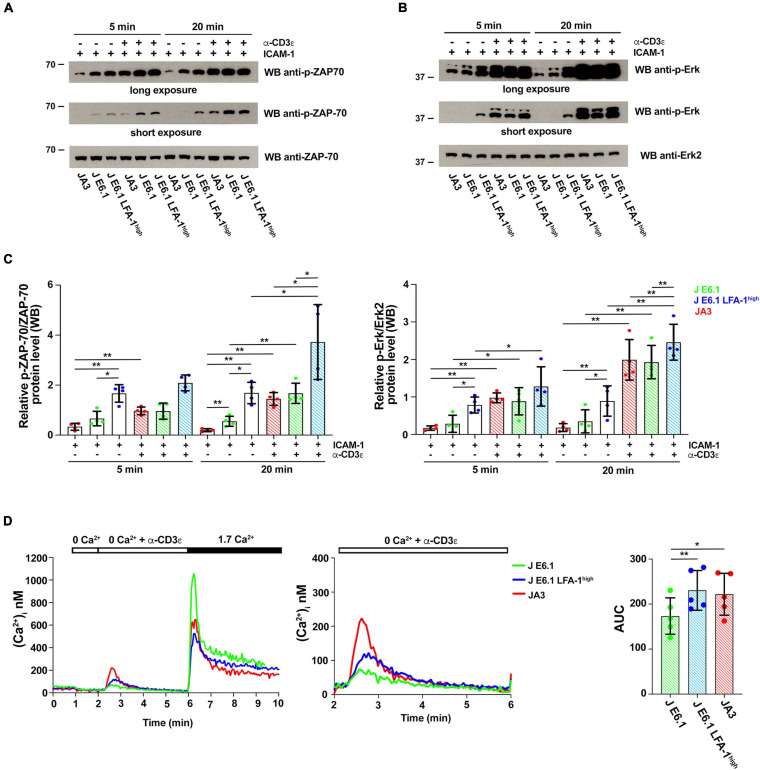
Increasing LFA-1 expression in E6.1 cells affects early signaling events. **(A,B)** Immunoblot analysis with anti-p-ZAP-70 **(A)** or anti-p-Erk1/2 **(B)** antibodies of lysates from E6.1, E6.1 LFA-1^*high*^ or JA3 Jurkat cells plated on glass-immobilized ICAM-1 in the presence or absence of anti-CD3ε mAb, clone OKT3 for 5 or 20 min. Anti-ZAP-70 and anti-Erk2 antibodies were used as respective loading controls. Representative blots are shown. The migration of molecular mass markers is indicated. **(C)** Quantification of the relative levels of p-ZAP-70 and p-Erk1/2, normalized to the respective loading control (mean ± SD; paired Student’s *t*-test; *n* = 4). Only significant differences are shown. *p < 0.05; **p < 0.01. **(D)** [Ca^2+^]_*i*_ mobilization in Fura-2/AM-loaded Jurkat cells. Cells were stimulated with anti-CD3ε mAb, clone OKT3 followed by anti-mouse IgG in Ca^2+^-free buffer to evaluate Ca^2+^ release followed by re-addition of 1.7 mM Ca^2+^ to evoke Ca^2+^ influx (left); a magnification of anti-CD3ε mAb (clone OKT3) + anti-mouse IgG-induced Ca^2+^ release is shown on the right. The histogram shows AUC values to quantify anti-CD3ε mAb-induced Ca^2+^ release (mean ± SD; paired Student’s *t*-test; *n* = 5). Only significant differences are shown. *p < 0.05; **p < 0.01.

To explore the outcome of the peculiar subcellular compartmentalization of tyrosine phosphoproteins in E6.1 LFA-1^*high*^ cells we carried out an immunoblot analysis on E6.1 and E6.1 LFA-1^*high*^ cells plated for 5 min or 20 min on glass-immobilized ICAM-1 in the presence or absence of anti-CD3ε mAb (clone OKT3). Interestingly, E6.1 LFA-1^*high*^ cells, either unstimulated or plated on ICAM-1 alone, had high basal levels of both p-ZAP-70 and p-Erk when compared to both E6.1 and JA3 cells (Figures 4A–C and Supplementary Figure 7). Signaling was enhanced in the presence of anti-CD3ε mAb at 5 min and further enhanced at 20 min in all cell lines (Figures 4A–C). Activated E6.1 LFA-1^*high*^ cells displayed significantly higher levels of p-ZAP-70 and p-Erk compared to E6.1 cells, suggesting that forced LFA-1 expression improves early signaling (Figures 4A–C; blue vs. green dot histograms). Interestingly, activated JA3 cells (red dot histograms) displayed lower levels of p-ZAP-70 and p-Erk in response to stimulation compared to the other cell lines, but also had the lowest levels of basal signaling (Figures 4A–C), making them the best responders.

To further investigate the potential impact of forced LFA-1 expression on early signaling in E6.1 cells, we measured Ca^2+^ mobilization from intracellular stores. Cells were loaded with the fluorescent Ca^2+^ indicator Fura-2/AM and Ca^2+^ flux was measured by fluorimetry either under resting conditions or following TCR cross-linking in Ca^2+^-free medium. The kinetics of intracellular Ca^2+^ mobilization was comparable between the two E6.1 cell lines, however total intracellular Ca^2+^ released over time was higher E6.1 LFA-1^*high*^ cells (Figure 4D). Total intracellular Ca^2+^ released over time was also higher in JA3 cells, but Ca^2+^ was released faster, reached a higher peak and returned rapidly to baseline compared to the E6.1 lines (Figure 4D, histogram), again highlighting JA3 cells as good responders.

**FIGURE 5:**
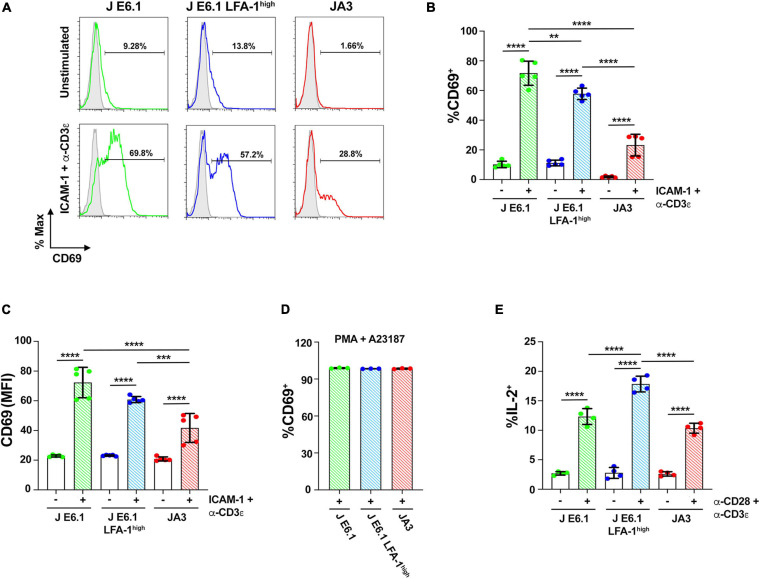
Increasing LFA-1 expression in E6.1 cells improves IL-2 expression. **(A)** Representative FACS profiles of CD69 staining in E6.1, E6.1 LFA-1^*high*^ or JA3 Jurkat cells either unstimulated or plated on glass-immobilized ICAM-1 and anti-CD3ε mAb, clone OKT3 for 16 h. **(B,C)** Histograms showing the percentage (%) of CD69^+^ cells **(B)** and CD69 MFI **(C)** among cells activated as in **(A)** (mean ± SD; One-Way ANOVA test; *n* ≥ 3). **(D)** Histogram showing the percentage (%) of CD69^+^ cells among JA3, E6.1 and E6.1 LFA-1^*high*^ Jurkat cells stimulated for 16 h with a combination of 100 ng/ml PMA and 500 ng/ml A23187 (mean ± SD; One-Way ANOVA test; *n* = 3). **(E)** Intracellular staining flow cytometry of E6.1, E6.1 LFA-1^*high*^ or JA3 Jurkat cells stimulated for 6 h on immobilized anti-CD3ε, clone OKT3 and anti-CD28 mAb. A cocktail of brefeldin A and monensin was added in the last 1 h of culture. Cells were permeabilized and stained with anti-IL-2 mAb and the percentage (%) of IL-2^+^ cells was quantified by flow cytometry. Only significant differences are shown. **p < 0.01; ***p < 0.001; ****p < 0.0001.

To assess how these differences in early signaling influence the downstream biological response, we carried out a flow cytometric analysis of the activation marker CD69 on the three Jurkat lines activated under the same conditions for 16 h. CD69 expression in the two E6.1 lines showed a bimodal distribution, with a CD69^*low*^ and a CD69^*high*^ population, the former being larger in E6.1 LFA-1^*high*^ cells (Figures 5A,B). The mean fluorescence intensity of CD69 in the CD69^*high*^ population was however comparable between E6.1 and E6.1 LFA-1^*high*^ cells (Figure 5C), indicating that fewer E6.1 LFA-1^*high*^ cells responded to the stimulation compared to E6.1 cells, but that these cells were equally efficient in expressing CD69. Activated JA3 cells displayed a homogeneous distribution of CD69, and both the percentage of CD69^+^ cells and the mean fluorescence intensity of CD69 were lower compared to both E6.1 and E6.1 LFA-1^*high*^ cells (Figures 5A–C). Of note, unstimulated JA3 cells had a very low basal frequency of CD69^+^ cells compared to the two E6.1 lines, consistent with the low levels of basal signaling (Figure 5B). No difference was observed when cells were stimulated pharmacologically using a combination of the phorbol ester PMA and the calcium ionophore A23187, which bypass TCR signaling (Figure 5D), indicating that the intracellular signaling machinery downstream of the TCR is functional in all cell lines.

We extended the study to a later biological readout of T cell activation, namely IL-2 expression, which has more stringent requirements compared to CD69 (Testi et al., 1989). The frequency of IL-2 expressing cells activation was measured by intracellular staining flow cytometry. The frequency of IL-2^+^ cells was higher among E6.1 LFA-1^*high*^ cells compared to E6.1 cells, with the lowest frequency among JA3 cells (Figure 5E), suggesting a correlation between the levels of p-ZAP-70/p-Erk achieved in response to early signaling and the expression of IL-2.

## Discussion

Similar to all immortalized cell lines, Jurkat cells do not fully recapitulate the features of their primary CD4^+^ counterparts. The differences must be kept in mind when extrapolating concepts from the results obtained using this T cell model, and validation in primary T cells is mandatory. For example, loss-of-function mutations in the genes encoding the lipid phosphatases PTEN and SHIP-1 make Jurkat E6.1 cells unsuitable for studies on PI3 kinase signaling (Shan et al., 2000; Freeburn et al., 2002; Gioia et al., 2018). Additionally, because the cognate peptide-MHC ligand of the Jurkat TCR is unknown, activation must be carried out using surrogate ligands, namely agonistic anti-CD3 antibodies or SEE (Kappler et al., 1989). The signaling pathways triggered by these agonists reproduce closely, but not completely, the pathway triggered by peptide-MHC (Zamoyska, 2006). Nonetheless, Jurkat cells remain a robust tool easily amenable to genetic manipulation and biochemical studies that is extensively used to provide working hypotheses to test *ex vivo* on primary T cells or *in vivo* in genetically engineered mice.

Supported lipid bilayers have become established as a versatile tool to visualize T cell autonomous aspects of IS dynamics at nanometer to micrometer scales (Dustin, 2010). The SLB mimics an APC in many aspects, but the T cell alone drives the nanometer to micrometer scale process of microcluster and SMAC formation. Incorporating into the basic setting of a TCR ligand (pMHC or anti-CD3 Fab’), an integrin ligand (e.g., ICAM-1) and other molecules that bind co-stimulatory or co-inhibitory receptors has brought to light subdomain organization of the classic SMACs characterized by the segregation of specific receptors, as recently shown for CD2 and CD28 (Demetriou et al., 2020). While Jurkat cells have been widely exploited for studies of IS assembly in the setting of SEE-specific cell-cell conjugates, to date their use for imaging studies on SLBs has been limited. However, the results have demonstrated that, notwithstanding the less compact distribution of TCR clusters at the cSMAC and subtle differences in the actin cytoskeleton architecture compared to quiescent primary CD4^+^ T cells (Murugesan et al., 2016; Colin-York et al., 2019), this model can be exploited as a convenient toolkit to analyze the dynamics of protein segregation during IS formation (Kaizuka et al., 2007, 2009). Our data show that, by compensating for the low expression of LFA-1 in the most extensively used Jurkat T cell clone E6.1, it is possible to enhance the ability of these cells to form well-structured immune synapses. In particular, while both E6.1 cells and their LFA-1^*high*^ counterparts effectively segregate the TCR, LFA-1 and F-actin in the respective SMACs, LFA-1 enhances the compartmentalization of the TCR to the cSMAC. This finding recapitulates in human T cells the finding that LFA-1 is dispensable for cSMAC formation in OVA-specific mouse CD4^+^ cells (Graf et al., 2007). Hence engineered LFA-1^*high*^ Jurkat E6.1 cells are a valuable model to study the redistribution of signaling molecules during IS assembly in the context of LFA-1 signaling and LFA-1-mediated adhesion using the SLB setting. The improvement in IS architecture associated with increased LFA-1 expression in E6.1 may impact also effector function requiring directed secretion of soluble factors (Huse et al., 2006) or extracellular vesicle budding (Choudhuri et al., 2014). Similarly, expression of CD40L in E6.1 cells could be studied not only in terms of transcription and surface expression, but also in terms of directed budding of TCR and CD40L positive synaptic ectosomes in the cSMAC (Saliba et al., 2019).

Surprisingly, while increasing LFA-1 expression in E6.1 cells improved their performance in the SLB system, neither conjugate formation nor pTyr signaling was enhanced in E6.1 LFA-1^*high*^ cells stimulated with SEE-pulsed APCs. This suggests that E6.1 cells may exploit other integrins or accessory surface receptors for TCR co-stimulation to compensate for the low levels of LFA-1, and indeed we found that the efficiency and extent of tyrosine phosphoprotein accumulation in E6.1 cells, but not in E6.1 LFA-1^*high*^ cells, relies in part on CD2 (unpublished results). Intriguingly, E6.1 LFA-1^*high*^ cells show a peculiar pattern of tyrosine phosphoprotein distribution at the IS, with a lower accumulation at the synaptic membrane compared to E6.1 cells concomitant with the presence of an intracellular pool that colocalizes with endosomal CD3ζ. This pool was present also in conjugates formed in the absence of SEE, albeit under these conditions it was not polarized at the T-cell:APC contact. This observation suggests that E6.1 LFA-1^*high*^ cells may have some constitutive activation due to enhanced TCR tonic signaling. This is supported by the enhanced basal activation of ZAP-70 and Erk observed in these cells. A basal endosomal accumulation of signaling-competent tyrosine-phosphorylated CD3ζ in Jurkat cells has been previously reported (Yudushkin and Vale, 2010). Although no information of LFA-1 expression was included in this study, we speculate that the Jurkat clone used may have been LFA-1^*high*^.

Interestingly, when comparing specific steps of the TCR signaling cascade in E6.1 and E6.1 LFA-1^*high*^ cells, forced LFA-1 expression showed an enhancing effect on the signaling readouts used, with an increase in the levels of p-ZAP-70 and p-Erk in response to activation and an increase in intracellular Ca^2+^ mobilization. This correlates with a higher expression of a late activation marker, IL-2, while expression of CD69 among responder cells was comparable. We can speculate that the signals elicited by the TCR in this setting are sufficient to activate maximal CD69 expression in E6.1 cells independently of the levels of surface LFA-1. This may not apply to IL-2 expression, which has more stringent requirements compared to CD69 (Testi et al., 1989). It is noteworthy that E6.1 LFA-1^*high*^ cells show a clear bimodal pattern of CD69 expression, with a substantial increase in the CD69-negative population. We could hypothesize that the high basal ZAP-70 and Erk activation in E6.1 LFA-1^*high*^ cells makes them partially anergic, such that not all cells are able to respond.

Interestingly, our results show that the JA3 clone, which was derived from the same Jurkat line as E6.1 cells (Moretta et al., 1985), has unique features compared to both E6.1 and E6.1 LFA-1^*high*^ cells. Despite expressing LFA-1 at levels comparable to E6.1 cells, JA3 cells form well-structured synapses displaying a tight segregation of the TCR to the cSMAC. Differences can also be detected in early signaling, with lower levels of p-ZAP-70 and p-Erk in response to activation compared to the two E6.1 lines but also very low basal signaling, which makes them better responders. Additionally, the kinetics of their Ca^2+^ response is faster. Nevertheless, similar to the E6.1 lines, the levels of CD69 and IL-2 expression in activated JA3 cells appear to correlate with the amount of activated p-ZAP-70 and p-Erk, making them the weakest CD69 and IL-2 expressers among the three Jurkat lines analyzed. These differences may be accounted for in part by the specific constellation of mutations accumulated in the selection process of the E6.1 and JA3 clones.

## Conclusion

**FIGURE 6:**
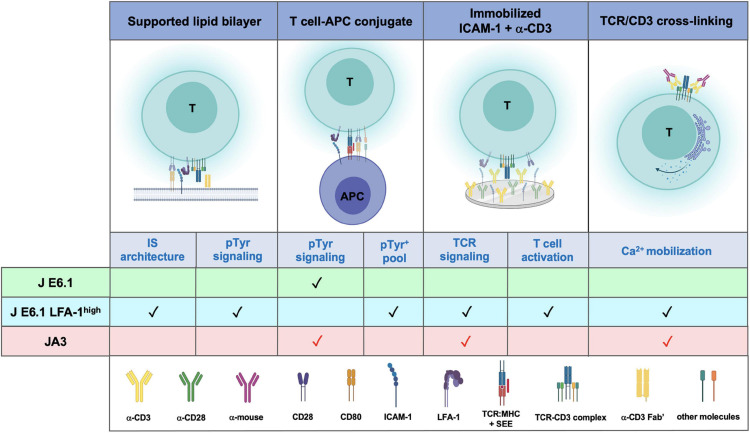
Comparative performance of E6.1, E6.1 LFA-1^*high*^ and JA3 cells in IS architecture, TCR signaling and T-cell activation. Schematic representation of experimental settings used for testing the performance of the three Jurkat lines. The symbol “✓” indicates the best responder between E6.1 and E6.1 LFA-1^*high*^ cells. The symbol “
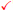
” indicates experimental settings in which JA3 cells are good responders. The figure was generated with BioRender.com.

Our data extend the exploitability of Jurkat E6.1 cells by providing a strategy to make them more amenable to imaging IS formation in the SLB setting. This would potentially allow reduction of animal use and further reduce the need for primary T cells in studies on IS architecture and its abnormalities in disease. The improvement in IS architecture of E6.1 cells resulting from forced LFA-1 expression entails, however, an enhancement in basal signaling. JA3 cells, which have the “cleanest” background, could complement E6.1 cells for signaling studies to fully exploit the Jurkat paradigm of T cell signaling (Figure 6).

## Data Availability Statement

The raw data supporting the conclusions of this article will be made available by the authors, without undue reservation.

## Author Contributions

All the authors listed have made a substantial, direct and intellectual contribution to the work, and approved it for publication. CC, SB, EBC, JHF, AG, CDB, SLF, and JB performed the experimental work. CC and SB prepared the figures. SV, DP, MMD’E, and LM contributed essential reagents.

## Conflict of Interest

The authors declare that the research was conducted in the absence of any commercial or financial relationships that could be construed as a potential conflict of interest.

## Publisher’s Note

All claims expressed in this article are solely those of the authors and do not necessarily represent those of their affiliated organizations, or those of the publisher, the editors and the reviewers. Any product that may be evaluated in this article, or claim that may be made by its manufacturer, is not guaranteed or endorsed by the publisher.

## References

[B1] Abraham R. T. Weiss A. (2004). Jurkat T cells and development of the T-cell receptor signalling paradigm. *Nat. Rev. Immunol.* 4 301–308. 10.1038/nri1330 15057788

[B2] Alcover A. Alberini C. Acuto O. Clayton L. K. Transy C. Spagnoli G. C. (1988). Interdependence of CD3-Ti and CD2 activation pathways in human T lymphocytes. *EMBO J.* 7, 1973-1977. 2901344 10.1002/j.1460-2075.1988.tb03035.x PMC454469

[B3] Blanchard N. Di Bartolo V. Hivroz C. (2002). In the immune synapse, ZAP-70 controls T cell polarization and recruitment of signaling proteins but not formation of the synaptic pattern. *Immunity* 17 389–399. 10.1016/s1074-7613(02)00421-1 12387734

[B4] Bunnell S. C. Hong D. I. Kardon J. R. Yamazaki T. Mcglade C. J. Barr V. A. (2002). T cell receptor ligation induces the formation of dynamically regulated signaling assemblies. *J. Cell Biol.* 158 1263–1275. 10.1083/jcb.200203043 12356870 PMC2173229

[B5] Bunnell S. C. Kapoor V. Trible R. P. Zhang W. Samelson L. E. (2001). Dynamic actin polymerization drives T cell receptor-induced spreading: a role for the signal transduction adaptor LAT. *Immunity* 14 315–329. 10.1016/s1074-7613(01)00112-1 11290340

[B6] Carrasco Y. R. Fleire S. J. Cameron T. Dustin M. L. Batista F. D. (2004). LFA-1/ICAM-1 interaction lowers the threshold of B cell activation by facilitating B cell adhesion and synapse formation. *Immunity* 20 589–599. 10.1016/s1074-7613(04)00105-0 15142527

[B7] Chen B. M. Al-Aghbar M. A. Lee C. H. Chang T. C. Su Y. C. Li Y. C. (2017). The Affinity of Elongated Membrane-Tethered Ligands Determines Potency of T Cell Receptor Triggering. *Front. Immunol.* 8:793. 10.3389/fimmu.2017.00793 28740495 PMC5502409

[B8] Choudhuri K. Llodra J. Roth E. W. Tsai J. Gordo S. Wucherpfennig K. W. (2014). Polarized release of T-cell-receptor-enriched microvesicles at the immunological synapse. *Nature* 507 118–123. 10.1038/nature12951 24487619 PMC3949170

[B9] Colin-York H. Kumari S. Barbieri L. Cords L. Fritzsche M. (2019). Distinct actin cytoskeleton behaviour in primary and immortalised T-cells. *J. Cell Sci.* 133:jcs232322. 31413071 10.1242/jcs.232322 PMC6898998

[B10] Danielian S. Alcover A. Polissard L. Stefanescu M. Acuto O. Fischer S. (1992). Both T cell receptor (TcR)-CD3 complex and CD2 increase the tyrosine kinase activity of p56lck. CD2 can mediate TcR-CD3-independent and CD45-dependent activation of p56lck. *Eur. J. Immunol.* 22, 2915-2921. 10.1002/eji.1830221124 1358625

[B11] Demetriou P. Abu-Shah E. Valvo S. Mccuaig S. Mayya V. Kvalvaag A. (2020). A dynamic CD2-rich compartment at the outer edge of the immunological synapse boosts and integrates signals. *Nat. Immunol.* 21 1232–1243. 10.1038/s41590-020-0770-x 32929275 PMC7611174

[B12] Dustin M. L. (2010). Insights into function of the immunological synapse from studies with supported planar bilayers. *Curr. Top. Microbiol. Immunol.* 340 1–24. 10.1007/978-3-642-03858-7_1 19960306

[B13] Dustin M. L. Colman D. R. (2002). Neural and immunological synaptic relations. *Science* 298 785–789. 10.1126/science.1076386 12399580

[B14] Dustin M. L. Springer T. A. (1988). Lymphocyte function-associated antigen-1 (LFA-1) interaction with intercellular adhesion molecule-1 (ICAM-1) is one of at least three mechanisms for lymphocyte adhesion to cultured endothelial cells. *J. Cell Biol.* 107, 321–331. 10.1083/jcb.107.1.321 3134364 PMC2115164

[B15] Felce J. H. Parolini L. Sezgin E. Cespedes P. F. Korobchevskaya K. Jones M. (2020). Single-Molecule, Super-Resolution, and Functional Analysis of G Protein-Coupled Receptor Behavior Within the T Cell Immunological Synapse. *Front. Cell Dev. Biol.* 8:608484. 10.3389/fcell.2020.608484 33537301 PMC7848080

[B16] Felce S. L. Farnie G. Dustin M. L. Felce J. H. (2021). RNA-Seq analysis of early transcriptional responses to activation in the leukaemic Jurkat E6.1 T cell line [version 2; peer review: 2 approved with reservations]. *Wellcome Open Res.* 5:42. 10.12688/wellcomeopenres.15748.2 PMC9971649 36865034

[B17] Finetti F. Paccani S. R. Riparbelli M. G. Giacomello E. Perinetti G. Pazour G. J. (2009). Intraflagellar transport is required for polarized recycling of the TCR/CD3 complex to the immune synapse. *Nat. Cell Biol.* 11 1332–1339. 10.1038/ncb1977 19855387 PMC2837911

[B18] Freeburn R. W. Wright K. L. Burgess S. J. Astoul E. Cantrell D. A. Ward S. G. (2002). Evidence that SHIP-1 contributes to phosphatidylinositol 3,4,5-trisphosphate metabolism in T lymphocytes and can regulate novel phosphoinositide 3-kinase effectors. *J. Immunol.* 169 5441–5450. 10.4049/jimmunol.169.10.5441 12421919

[B19] Fritzsche M. Fernandes R. A. Chang V. T. Colin-York H. Clausen M. P. Felce J. H. (2017). Cytoskeletal actin dynamics shape a ramifying actin network underpinning immunological synapse formation. *Sci. Adv.* 3:e1603032. 10.1126/sciadv.1603032 28691087 PMC5479650

[B20] Gamberucci A. Innocenti B. Fulceri R. Banhegyi G. Giunti R. Pozzan T. (1994). Modulation of Ca2+ influx dependent on store depletion by intracellular adenine-guanine nucleotide levels. *J. Biol. Chem.* 269 23597–23602. 10.1016/s0021-9258(17)31557-0 8089128

[B21] Gillis S. Watson J. (1980). Biochemical and biological characterization of lymphocyte regulatory molecules. V. Identification of an interleukin 2-producing human leukemia T cell line. *J. Exp. Med.* 152, 1709–1719. 10.1084/jem.152.6.1709 6778951 PMC2186024

[B22] Gioia L. Siddique A. Head S. R. Salomon D. R. Su A. I. (2018). A genome-wide survey of mutations in the Jurkat cell line. *BMC Genomics* 19:334. 10.1186/s12864-018-4718-6 29739316 PMC5941560

[B23] Graf B. Bushnell T. Miller J. (2007). LFA-1-mediated T cell costimulation through increased localization of TCR/class II complexes to the central supramolecular activation cluster and exclusion of CD45 from the immunological synapse. *J. Immunol.* 179 1616–1624. 10.4049/jimmunol.179.3.1616 17641028 PMC3993012

[B24] Grakoui A. Bromley S. K. Sumen C. Davis M. M. Shaw A. S. Allen P. M. (1999). The immunological synapse: a molecular machine controlling T cell activation. *Science* 285 221–227. 10.1126/science.285.5425.221 10398592

[B25] Harburger D. S. Calderwood D. A. (2009). Integrin signalling at a glance. *J. Cell Sci.* 122 159–163. 10.1242/jcs.018093 19118207 PMC2714413

[B26] Huse M. Lillemeier B. F. Kuhns M. S. Chen D. S. Davis M. M. (2006). T cells use two directionally distinct pathways for cytokine secretion. *Nat. Immunol.* 7 247–255. 10.1038/ni1304 16444260

[B27] Kaizuka Y. Douglass A. D. Vardhana S. Dustin M. L. Vale R. D. (2009). The coreceptor CD2 uses plasma membrane microdomains to transduce signals in T cells. *J. Cell Biol.* 185 521–534. 10.1083/jcb.200809136 19398758 PMC2700390

[B28] Kaizuka Y. Douglass A. D. Varma R. Dustin M. L. Vale R. D. (2007). Mechanisms for segregating T cell receptor and adhesion molecules during immunological synapse formation in Jurkat T cells. *Proc. Natl. Acad. Sci. U. S. A.* 104 20296–20301. 10.1073/pnas.0710258105 18077330 PMC2154425

[B29] Kappler J. Kotzin B. Herron L. Gelfand E. W. Bigler R. D. Boylston A. (1989). V beta-specific stimulation of human T cells by staphylococcal toxins. *Science* 244 811–813. 10.1126/science.2524876 2524876

[B30] Kumari S. Depoil D. Martinelli R. Judokusumo E. Carmona G. Gertler F. B. (2015). Actin foci facilitate activation of the phospholipase C-γ in primary T lymphocytes via the WASP pathway. *Elife* 4:e04953. 10.7554/eLife.04953 PMC4355629 25758716

[B31] Manders E. M. Stap J. Brakenhoff G. J. Van Driel R. Aten J. A. (1992). Dynamics of three-dimensional replication patterns during the S-phase, analysed by double labelling of DNA and confocal microscopy. *J. Cell Sci.* 103 857–862. 10.1242/jcs.103.3.857 1478975

[B32] Mastrogiovanni M. Juzans M. Alcover A. Di Bartolo V. (2020). Coordinating Cytoskeleton and Molecular Traffic in T Cell Migration, Activation, and Effector Functions. *Front. Cell Dev. Biol.* 8:591348. 10.3389/fcell.2020.591348 33195256 PMC7609836

[B33] Mayya V. Judokusumo E. Abu Shah E. Peel C. G. Neiswanger W. Depoil D. (2018). Durable Interactions of T Cells with T Cell Receptor Stimuli in the Absence of a Stable Immunological Synapse. *Cell Rep.* 22 340–349. 10.1016/j.celrep.2017.12.052 29320731 PMC5775504

[B34] Moingeon P. Alcover A. Clayton L. K. Chang H. C. Transy C. Reinherz E. L. (1988). Expression of a functional CD3-Ti antigen/MHC receptor in the absence of surface CD2. Analysis with clonal Jurkat cell mutants. *J. Exp. Med.* 168, 2077–2090. 10.1084/jem.168.6.2077 3264323 PMC2189161

[B35] Monks C. R. Freiberg B. A. Kupfer H. Sciaky N. Kupfer A. (1998). Three-dimensional segregation of supramolecular activation clusters in T cells. *Nature* 395 82–86. 10.1038/25764 9738502

[B36] Moretta A. Pantaleo G. Lopez-Botet M. Moretta L. (1985). Involvement of T44 molecules in an antigen-independent pathway of T cell activation. Analysis of the correlations to the T cell antigen-receptor complex. *J. Exp. Med.* 162 823–838. 10.1084/jem.162.3.823 3875683 PMC2187795

[B37] Moretta A. Poggi A. Olive D. Bottino C. Fortis C. Pantaleo G. (1987). Selection and characterization of T-cell variants lacking molecules involved in T-cell activation (T3 T-cell receptor, T44, and T11): analysis of the functional relationship among different pathways of activation. *Proc. Natl. Acad. Sci. U.S.A.* 84, 1654–1658. 10.1073/pnas.84.6.1654 2951735 PMC304495

[B38] Murugesan S. Hong J. Yi J. Li D. Beach J. R. Shao L. (2016). Formin-generated actomyosin arcs propel T cell receptor microcluster movement at the immune synapse. *J. Cell Biol.* 215 383–399. 10.1083/jcb.201603080 27799367 PMC5100289

[B39] Norcross M. A. (1984). A synaptic basis for T-lymphocyte activation. *Ann. Immunol. (Paris).* 135D, 113–134. 10.1016/s0769-2625(84)81105-8 6151375 PMC2551763

[B40] Onnis A. Baldari C. T. (2019). Orchestration of Immunological Synapse Assembly by vesicular trafficking. *Front. Dev. Cell Biol.* 7:110. 10.3389/fcell.2019.00110 31334230 PMC6616304

[B41] Ritter A. T. Asano Y. Stinchcombe J. C. Dieckmann N. M. Chen B. C. Gawden-Bone C. (2015). Actin depletion initiates events leading to granule secretion at the immunological synapse. *Immunity* 42 864–876. 10.1016/j.immuni.2015.04.013 25992860 PMC4448150

[B42] Ritter A. T. Kapnick S. M. Murugesan S. Schwartzberg P. L. Griffiths G. M. Lippincott-Schwartz J. (2017). Cortical actin recovery at the immunological synapse leads to termination of lytic granule secretion in cytotoxic T lymphocytes. *Proc. Natl. Acad. Sci. U. S. A.* 114 E6585–E6594. 28716933 10.1073/pnas.1710751114 PMC5559056

[B43] Rubin B. Llobera R. Gouaillard C. Alcover A. Arnaud J. (2000). Dissection of the role of CD3gamma chains in profound but reversible T-cell receptor down-regulation. *Scand. J. Immunol.* 52, 173–183. 10.1046/j.1365-3083.2000.00767.x 10931385

[B44] Saliba D. G. Cespedes-Donoso P. F. Balint S. Compeer E. B. Korobchevskaya K. Valvo S. (2019). Composition and structure of synaptic ectosomes exporting antigen receptor linked to functional CD40 ligand from helper T cells. *Elife* 8:e47528. 31469364 10.7554/eLife.47528 PMC6748831

[B45] Schneider U. Schwenk H. U. Bornkamm G. (1977). Characterization of EBV-genome negative “null” and “T” cell lines derived from children with acute lymphoblastic leukemia and leukemic transformed non-Hodgkin lymphoma. *Int. J. Cancer* 19, 621–626. 10.1002/ijc.2910190505 68013

[B46] Shan X. Czar M. J. Bunnell S. C. Liu P. Liu Y. Schwartzberg P. L. (2000). Deficiency of PTEN in Jurkat T cells causes constitutive localization of Itk to the plasma membrane and hyperresponsiveness to CD3 stimulation. *Mol. Cell Biol.* 20 6945–6957. 10.1128/mcb.20.18.6945-6957.2000 10958690 PMC88770

[B47] Testi R. Phillips J. H. Lanier L. L. (1989). T cell activation via Leu-23 (CD69). *J. Immunol.* 143, 1123–1128. 2501389

[B48] Varma R. Campi G. Yokosuka T. Saito T. Dustin M. L. (2006). T cell receptor-proximal signals are sustained in peripheral microclusters and terminated in the central supramolecular activation cluster. *Immunity* 25 117–127. 10.1016/j.immuni.2006.04.010 16860761 PMC1626533

[B49] Weiss A. Wiskocil R. L. Stobo J. D. (1984). The role of T3 surface molecules in the activation of human T cells: a two-stimulus requirement for IL 2 production reflects events occurring at a pre-translational level. *J. Immunol.* 133 123–128. 6327821

[B50] Yudushkin I. A. Vale R. D. (2010). Imaging T-cell receptor activation reveals accumulation of tyrosine-phosphorylated CD3ζ in the endosomal compartment. *Proc. Natl. Acad. Sci. U. S. A.* 107 22128–22133. 10.1073/pnas.1016388108 21135224 PMC3009781

[B51] Zamoyska R. (2006). Superantigens: supersignalers? *Sci Stke* 2006:e45. 10.1126/stke.3582006pe45 17062896

