# Supplementary material for: Increasing LFA-1 Expression Enhances Immune Synapse Architecture and T Cell Receptor Signaling in Jurkat E6.1 Cells
Source: Front Cell Dev Biol. 2021 Jul 23;9:673446. doi: 10.3389/fcell.2021.673446 (PMC8343233; doi:10.3389/fcell.2021.673446)
Supplement: Supplementary file 7 [file Data_Sheet_1.pdf]

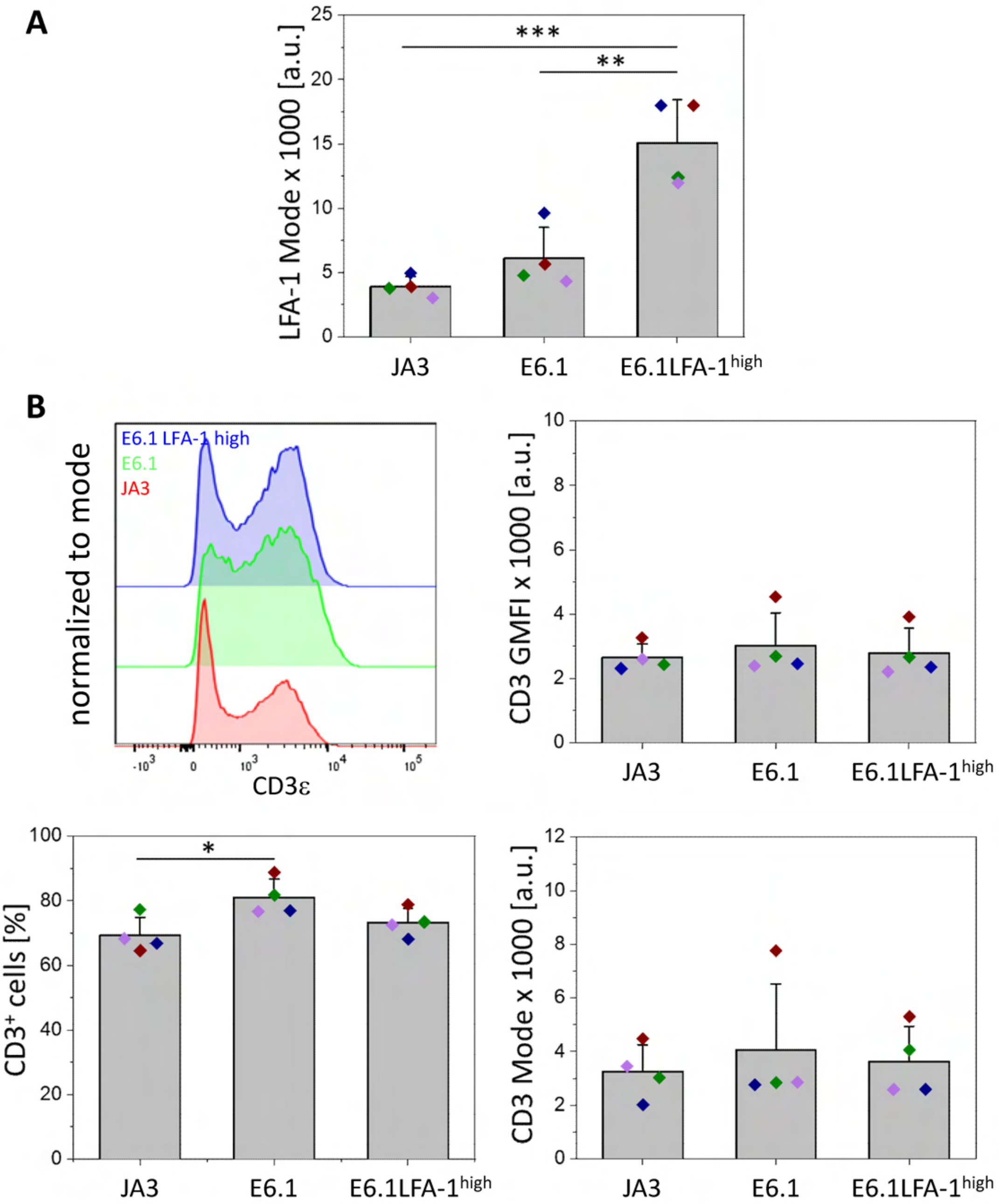
A
LFA-1 Mode x 1000 [a.u.]
25
20
15
10
5
0
***
**
JA3
E6.1
E6.1LFA-1high
B
E6.1 LFA-1 high
E6.1
JA3
normalized to mode
CD3ε
CD3 GMFI x 1000 [a.u.]
CD3+ cells [%]
CD3 Mode x 1000 [a.u.]

FIGURE S1

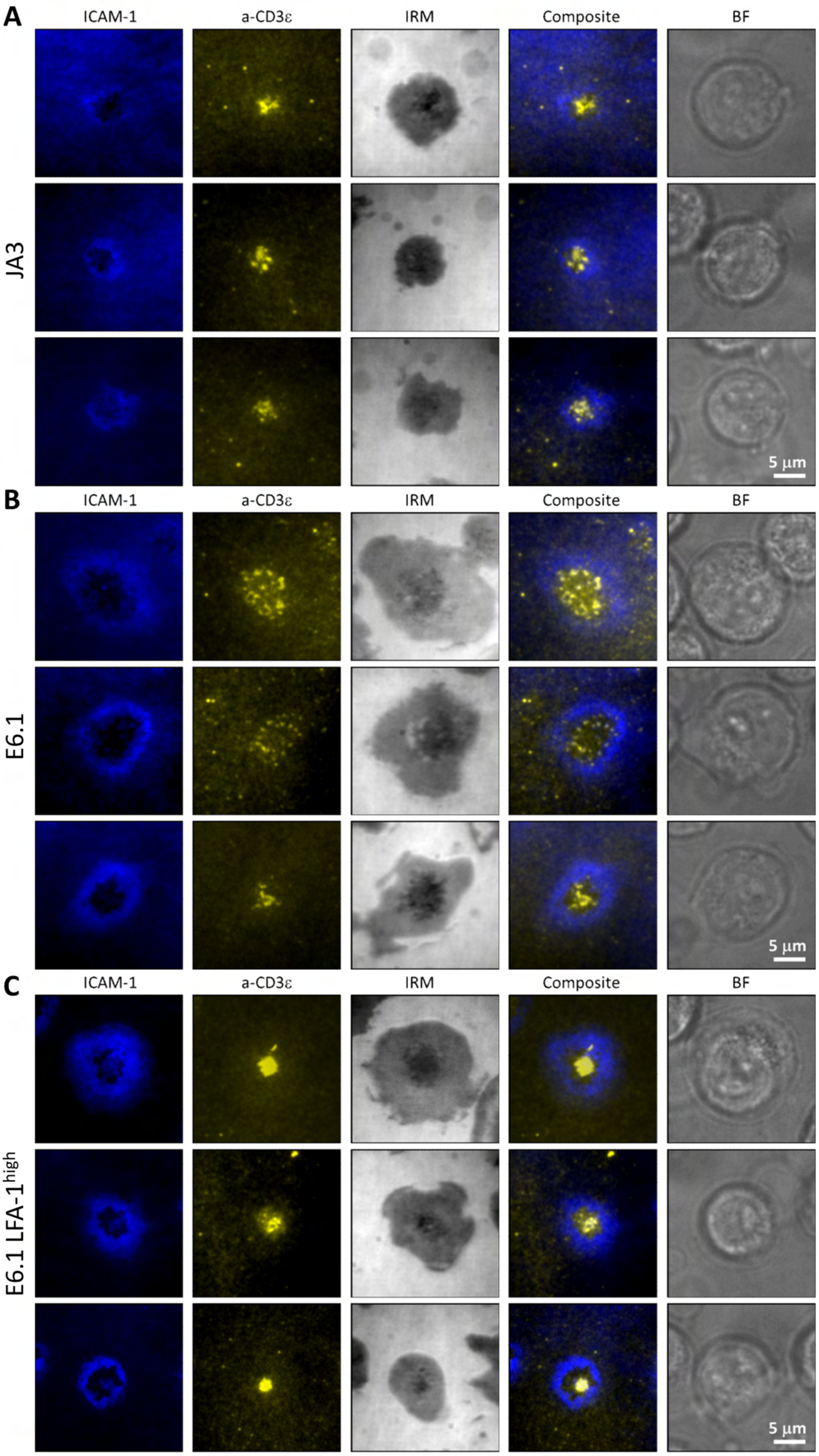
A
ICAM-1
a-CD3ε
IRM
Composite
BF
JA3
5 μm
B
ICAM-1
a-CD3ε
IRM
Composite
BF
E6.1
5 μm
C
ICAM-1
a-CD3ε
IRM
Composite
BF
E6.1 LFA-1high
5 μm

FIGURE S2

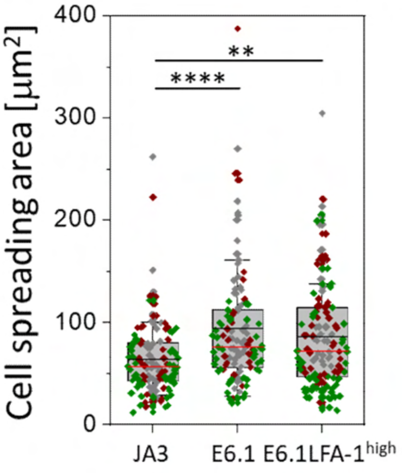
Cell spreading area [µm2]
400
300
200
100
0
**
****
JA3
E6.1
E6.1LFA-1high

FIGURE S3

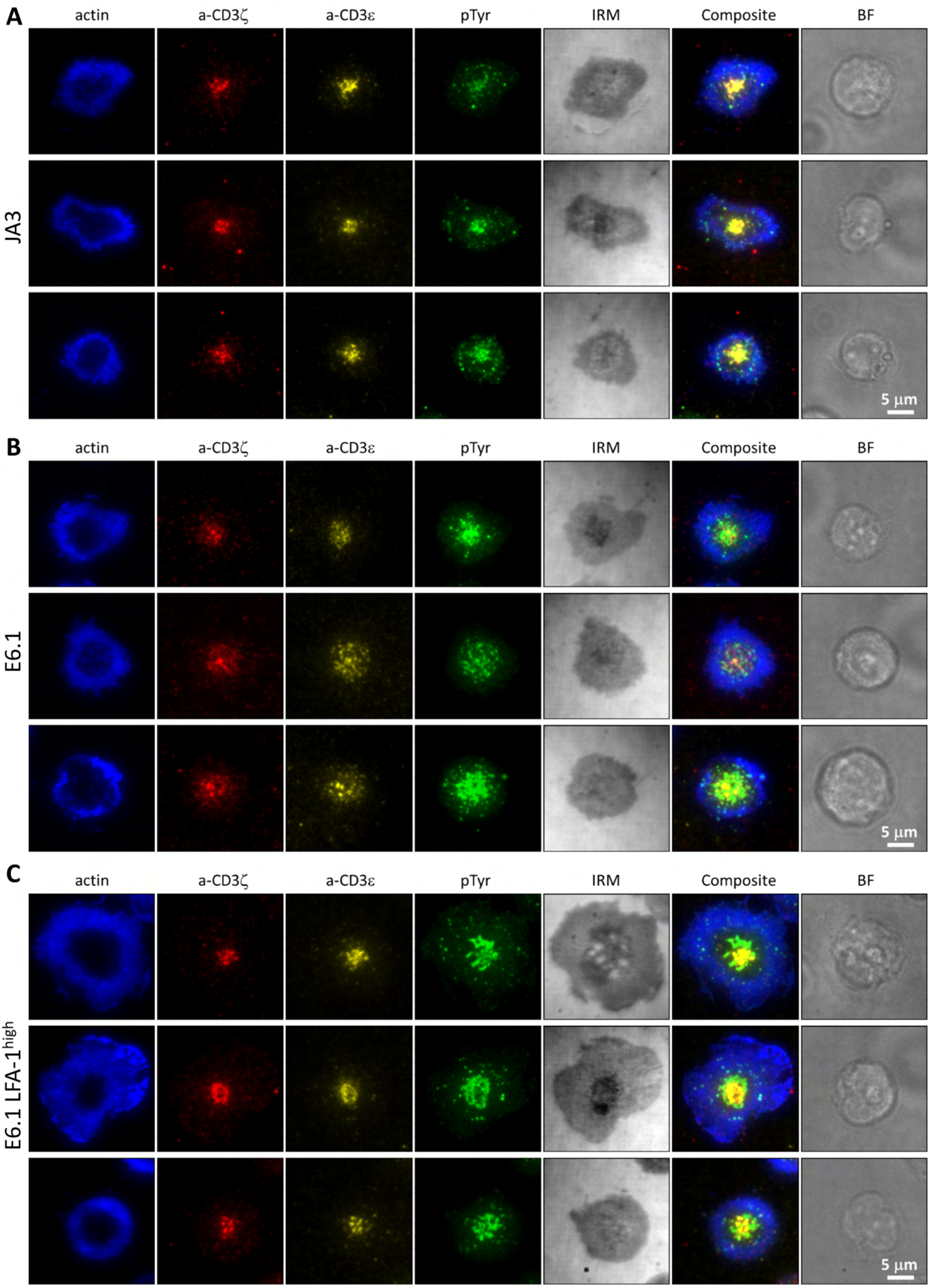
A
actin
a-CD3ζ
a-CD3ε
pTyr
IRM
Composite
BF
JA3
5 µm
B
E6.1
C
E6.1 LFA-1high

FIGURE S4

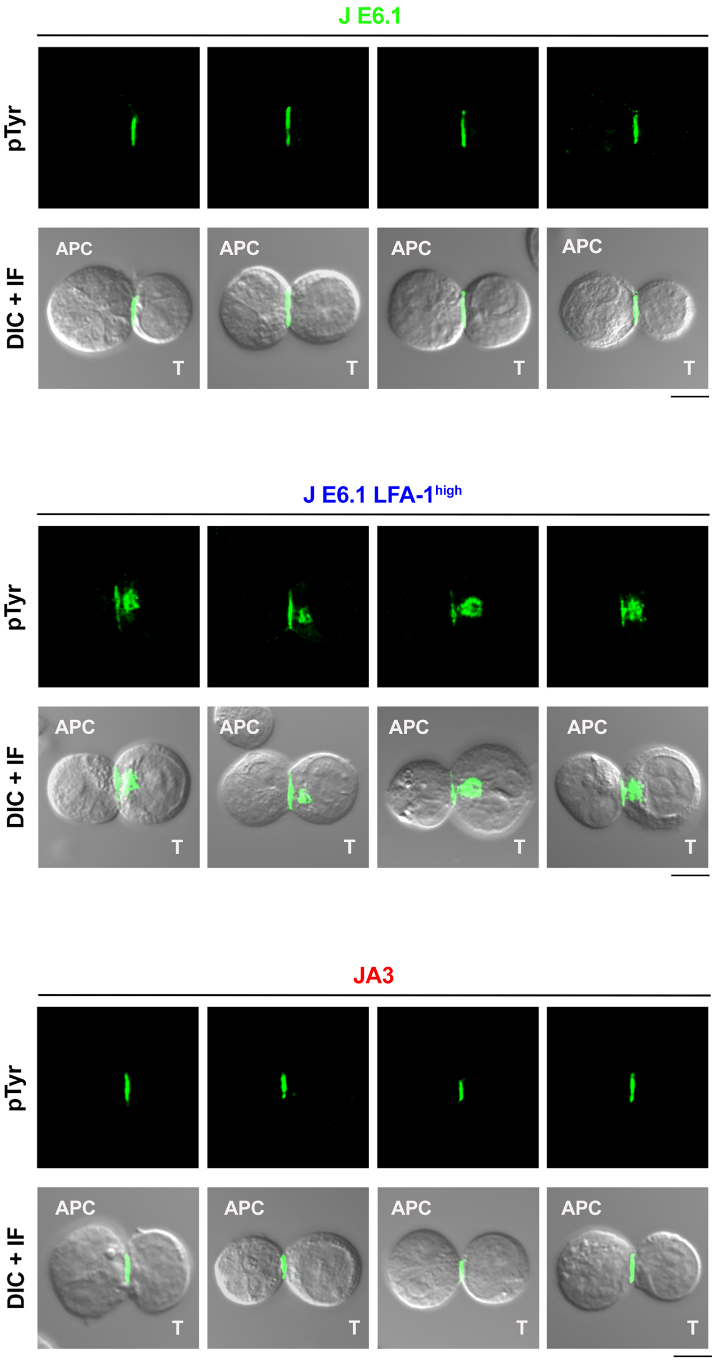
J E6.1
pTyr
DIC + IF
APC
T
J E6.1 LFA-1high
pTyr
DIC + IF
APC
T
JA3
pTyr
DIC + IF
APC
T

FIGURE S5

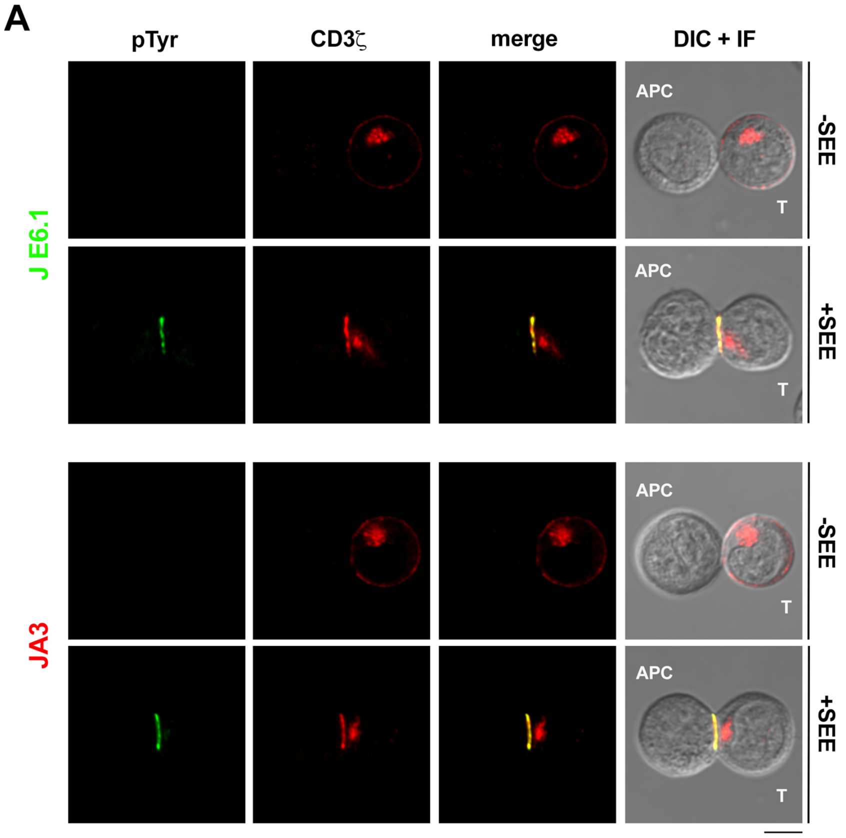

A
pTyr
CD3ζ
merge
DIC + IF
J E6.1
JA3
APC
T
-SEE
+SEE

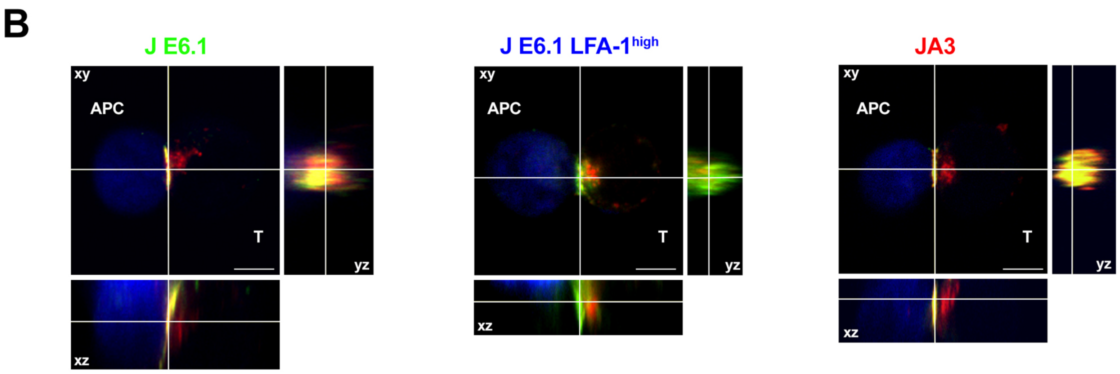

B
J E6.1
J E6.1 LFA-1high
JA3
xy
yz
xz
APC
T

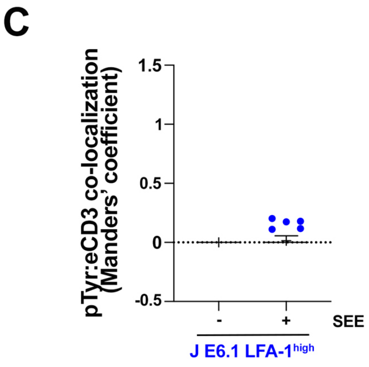

C
pTyr:eCD3 co-localization (Manders' coefficient)
1.5
1
0.5
0
-0.5
-
+
SEE
J E6.1 LFA-1high

FIGURE S6

A

B

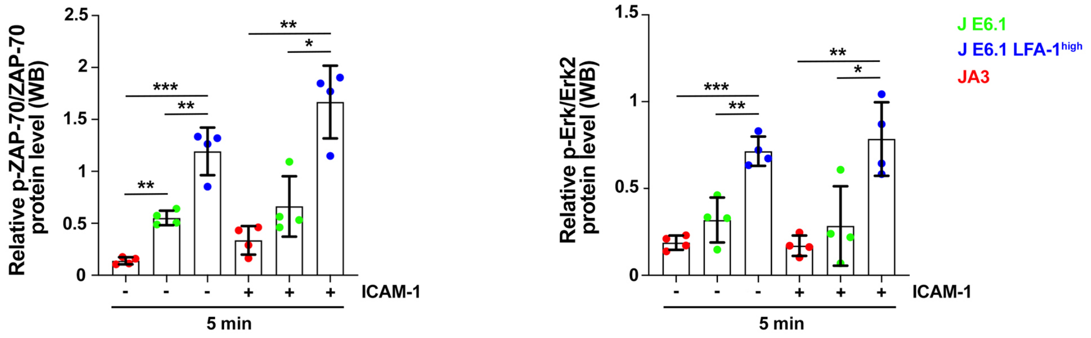
Relative p-ZAP-70/ZAP-70 protein level (WB)
2.5
2
1.5
1
0.5
0
**
***
**
**
*
- - - + + + ICAM-1
5 min
Relative p-Erk/Erk2 protein level (WB)
1.5
1
0.5
0
***
**
**
*
- - - + + + ICAM-1
5 min
J E6.1
J E6.1 LFA-1high
JA3

FIGURE S7

SUPPLEMENTAL FIGURE LEGENDS

Figure S1. Jurkat cell lines express comparable surface levels of CD3ε. A. Flow cytometric analysis of the surface expression of LFA-1 on JA3, E6.1 and E6.1 LFA-1^{high} Jurkat cells. The histograms represent Mode of LFA-1. **B.** Flow cytometric analysis of the surface expression of CD3ε on JA3, E6.1 and E6.1 LFA-1^{high} Jurkat cells. Representative FACS plots of CD3ε are shown. The histograms represent GMFI, Mode and percentage of CD3ε. Bars and error bars represent mean±SD. Data are from 4 independent experiments; each dot and colour represents an independent experiment. One-way analysis of variance (ANOVA) with Tukey's post-hoc test. Only significant differences are shown. *, p<0.05; **, p<0.01; ***, p<0.001.

Figure S2. Representative TIRFM images of JA3 (**A**), E6.1 (**B**) or E6.1 LFA-1^{high} (**C**) Jurkat cells interacting with activating [ICAM-1-AF405 (blue) + anti-CD3ε UCHT1-CF568 Fab' (yellow)] SLB for 15 min. IRM – Interference reflection microscopy, BF – Bright Field. Scale bar, 5 µm.

Figure S3. Quantification of the cell spreading area of JA3, E6.1 and E6.1 LFA-1^{high} Jurkat cells interacting with activating SLB for 15 min. Horizontal lines and error bars represent mean±SD (black line) and median (red line). Gray boxes represent 25-75 percentile. Data are from minimum of 120 cells from 3 independent experiments; each dot represents a cell; each colour represents an independent experiment. One-way analysis of variance (ANOVA) with Tukey's post-hoc test. Only significant differences are shown. **, p<0.01; ****, p<0.0001.

Figure S4. Representative TIRFM images of JA3 (**A**), E6.1 (**B**) or E6.1 LFA-1^{high} (**C**) Jurkat cells interacting with activating [ICAM-1 + anti-CD3ε UCHT1-CF568 Fab' (yellow)] SLB for 15 min. Cells were permeabilized and stained with directly conjugated primary antibodies against anti-CD3ζ-AF647 (red), anti-phosphotyrosine pTyr-AF488 (green) and the cell actin cytoskeleton was labelled with phalloidin-AF405 (blue). IRM – Interference reflection microscopy, BF – Bright Field. Scale bar, 5 µm.

Figure S5. Gallery of examples of SEE-specific conjugates of E6.1, E6.1 LFA-1^{high} and JA3 Jurkat cells. Representative images of SEE-specific conjugates stained with anti-pTyr antibody as described for the representative images in figure 3.

Figure S6. Neither E6.1 nor JA3 cells have an intracellular pTyr pool associated with endosomal CD3ζ, as opposed to E6.1 LFA-1^{high} cells. A. Immunofluorescence analysis of 15-min conjugates of E6.1 or JA3 cells and Raji cells (APC) in the presence or absence of SEE. Cells were co-stained with anti-pTyr and anti-CD3ζ antibodies. The histogram showing the quantification of the co-localization of pTyr and the endosomal CD3ζ pool in E6.1 LFA-1^{high} conjugates is presented in figure 3E. **B.** Representative xy and orthogonal views of conjugates formed by E6.1, E6.1 LFA-1^{high} or JA3 with SEE-pulsed Raji B cells (3D reconstructions of representative z-stacks are shown in supplemental videos 1-3). Cells were co-stained with anti-pTyr and anti-CD3ζ antibodies. **C.** Control of co-localization specificity of pTyr and the endosomal CD3ζ pool in E6.1 LFA-1^{high} cells. Co-localization was measured after tilting one staining relative to the other of 90 degrees (mean±SD; Kruskal-Wallis test; n ≥ 20 conjugates/sample from 3 independent experiments). Graph boxes represent 10-90 percentile and the mean is shown as "+". Only significant differences are shown. Scale bar, 5 µm.

Figure S7. LFA-1 engagement does not enhance basal signaling in E6.1, E6.1 LFA-1^{high} or JA3 Jurkat cells. A,B. Immunoblot analysis with anti-p-ZAP-70 (**A**) or anti-p-Erk1/2 (**B**) antibodies of lysates from E6.1, E6.1 LFA-1^{high} or JA3 Jurkat cells either unstimulated or plated on glass-immobilized ICAM-1 for 5 min. Anti-ZAP-70 and anti-Erk2 antibodies were used as respective loading controls. The histograms show the quantification of the relative levels of p-ZAP-70 and p-Erk1/2, normalized to the respective loading controls (mean±SD; paired Student's *t*-test; n=4). Only significant differences are shown. * p<0.05; ** p<0.01; *** p<0.001.

Video 1_J E6.1. The movie shows a representative confocal *z*-stack of a E6.1-APC conjugate. CD3ε, red; pTyr, green; SEE-pulsed Raji, blue.

Video 2_J E6.1 LFA-1^{high}. The movie shows a representative confocal *z*-stack of a E6.1 LFA-1^{high}-APC conjugate. CD3ε, red; pTyr, green; SEE-pulsed Raji, blue.

Video 3_JA3. The movie shows a representative confocal *z*-stack of a JA3-APC conjugate. CD3ε, red; pTyr, green; SEE-pulsed Raji, blue.

Video 4_J E6.1. The movie shows a representative confocal *z*-stack and orthogonal view of the distribution of CD3ε and pTyr in E6.1 Jurkat cell line under activating conditions. CD3ε, red; pTyr, green; actin, blue.

Video 5_J E6.1 LFA-1^{high}. The movie shows a representative confocal *z*-stack and orthogonal view of the distribution of CD3ε and pTyr in E6.1 LFA-1^{high} Jurkat cell line under activating conditions. CD3ε, red; pTyr, green; actin, blue.

Video 5_JA3. The movie shows a representative confocal *z*-stack and orthogonal view of the distribution of CD3ε and pTyr in JA3 Jurkat cell line under activating conditions. CD3ε, red; pTyr, green; actin, blue.
